# Detecting level of wetland encroachment for urban agriculture in Uganda using hyper-temporal remote sensing

**DOI:** 10.12688/aasopenres.13040.1

**Published:** 2020-05-12

**Authors:** Stella Kabiri, Molly Allen, Juduth Toma Okuonzia, Beatrice Akello, Rebecca Ssabaganzi, Drake Mubiru

**Affiliations:** 1 Mukono Zonal Agricultural Research Institute (MUZARDI), National Agricultural Research Organisation, Mukono, P.O.Box. 164, Mukono, Uganda; 2 National Livestock Resource Research Institute (NARLRRI), National Agricultural Research Organization, Kampala, P. O. Box 5706, Uganda; 3 Facaulty of Natural Resources and Environmental Sciences, Busitema University, Tororo, P.O.236, Uganda; 4 Wakiso District Local Government, Department of Natural Resources, Wakiso, Uganda; 5 National Agricultural Research Laboratories (NARL) of the National Agricultural research Organization, Kampala, P.O.Box 7065, Uganda

**Keywords:** Environmental degradation, Papyrus wetlands, Lake Victoria, Urban growth, Sustainability

## Abstract

**Background: **Urbanization is an important indicator of economic growth and social change but is associated with environmental degradation. In Uganda, wetlands cover an area of 11% of the country’s land area, of which half have been converted to industry and residential areas, and urban agriculture. Here, we investigate the extent of wetlands lost in two Ugandan cities, Wakiso and Kampala, in a period of 30 years. Secondly, we demonstrate a simple methodology to monitor agriculture on encroached wetlands.

**Methods: **Using a field survey and free remote sensing data from Landsat TM 1986 and Landsat ETM 2016 we classified the rate of wetland loss and encroachment from 1986 to 2016. Using MODIS NDVI 16-day composites at 500-meter spatial resolution, we generated distinctive crops and crop mixtures in the encroached wetlands for urban agriculture using the ISODATA clustering algorithm.

**Results:** Over 30 years, 72,828 ha (73%) of the Wakiso-Kampala wetlands have been lost. Agriculture areas have doubled, of which 16,488 ha (23%) were reclaimed from wetlands. All cultivated agriculture in Kampala was in the wetlands while in Wakiso, 73% of crop agriculture was in the wetlands. Major crops grown in these urban wetlands were banana (20%), sugarcane (22%), maize (17%), *Eucalyptus* trees (12%), sweet potatoes (10%).

**Conclusions: **The Kampala-Wakiso wetlands have been disappearing at a rate of 2500 ha annually for the last 30 years. At this rate, there will be no wetlands left by 2029. Policy recommendations should promote wetland reclamation programs so as to restore and reconstruct lost and fragmented wetlands; should mandate food security and poverty eradication to convene with ministries regulating wetlands to merge conflicting policies; and should develop polices that are inclusive of challenges faced by the urban poor while at the same time minimize the pressures on urban environments.

## Introduction

While global urbanization is stipulated to increase to 67% by 2050, Africa’s urban population is predicted to triple ( UN-HABITAT, 2012). Although urbanization is an important indicator of economic growth and social change, this fast growth is associated with environmental degradation, which threatens sustainable growth of African cities. One of the most vulnerable ecosystems in urban areas are wetlands. Wetlands of the world cover 9% of the global land area ( Zedler & Kercher, 2005). Human induced activities driven by population pressure, expansion of agricultural land area, land degradation and poor policies have led to the loss of at least 50% of the global wetland land area ( Chapman *et al.*, 2001; Hartter & Southworth, 2009; IUCN, 1996). As a result ecosystem services performed by wetlands, such as water quality improvement, flood abatement, carbon sequestration, biodiversity ecological units of wild life and medicinal plants, have been reduced ( Dugan, 1993; Joosten, 2009; Saunders *et al.*, 2012; Schuyt 2005b; Zedler & Kercher, 2005).

In Uganda, wetlands cover an area of 11% of the country’s land area, with seasonal wetlands covering 7.7%, while permanent wetlands and swamp forests cover 3.4% and 0.1%, respectively (e.g. WETLANDS-ATLAS, 2016). A recent study in Kampala by Abebe (2013) showed that 658 hectares of permanent wetlands in Kampala, Uganda’s capital, had been converted to built-up areas between 1989 and 2010. However, there exists limited information on the extent of wetland conversion or utilization for urban agriculture. There is evidence that former rural farmers who migrate to urban areas transfer rural livelihood strategies by engaging in urban agriculture, which is most often in the wetlands ( Isunju *et al.*, 2016a). Wetland encroachment has increasingly become hazardous to the most vulnerable urban poor whose livelihoods depend on their immediate environment ( African Development Fund, 2008; Isunju *et al.*, 2016b). In spite of the hazards, food security of livelihoods living around wetlands is supported by abundant soil moisture and fertile sediments used for crop farming almost throughout the year ( Turyahabwe *et al.*, 2013). Despite the importance and value of these services for many people, wetlands are also amongst the most threatened ecosystems globally, especially from the effects of agriculture ( Falkenmark *et al.*, 2007; Finlayson, 2010). In sub-Saharan Africa, policy makers face a dilemma of policy regulations with wetlands, as they support the livelihoods of many poor people through the provision of numerous ecosystem services, including food ( Bikangaga *et al.*, 2007).

Uganda has seven policies that emphasize optimization of sustainable benefits of wetlands, while conserving the environment and biodiversity. These policies include The National Policy for the Conservation and Management of Wetlands of 1995, the National Environment Act of 1995, the Land Act of 1997, the Local Government Act of 1997, the Environment Impact Assessment Regulations of 1998, the Wetland Regulations of 2000, and the Constitution of 2010 ( WETLANDS-ATLAS, 2016). These policies emphasise protection of wetlands and forbid any form of wetland reclamation ( Isunju *et al.*, 2016a). They are enforced by the National Environmental Management Authority (NEMA), the Kampala Capital City Authority (KCCA) and the Ministry of Water and Environment, who call for eviction of wetland encroachers ( Isunju *et al.*, 2016a; MWE, 2001). Nevertheless, despite these policy interventions, half of the wetland areas in Ugandan cities have been converted to industry and residential areas, and crop land ( MWE, 2014; UBOS, 2009). The presidential initiative of Operation Wealth Creation ( OWC, 2017) and Uganda’s Vision 2040 policies ( NDPII, 2015) include increasing the ability of the poor to raise incomes and improve the quality of life of the poor. Wetlands in Ugandan cities are a key source of livelihood for the urban poor and yet over exploitation can lead to land degradation and risk of food shortages. This implies that there lies a dilemma in implementing these wetland conservation policies in the same framework as Operation Wealth Creation ( OWC, 2017), Sustainable Development Goal 11 (Sustainable cities and communities) and Uganda’s Vision 2040 policy ( NDPII, 2015), in regards to urban areas.

Twenty years ago, 35% of Kampala households engaged in agriculture within the city ( Maxwell, 1995). Agriculture land in Kampala comprised of a total of 11, 942 hectares which was 56.1% of the total land area of the city ( Maxwell, 1995). A recent study observed that currently the population of Kampala engaged in agriculture has dropped to 5.1%, and yet 38% of household income was from crop production ( UBOS, 2016). In another urban district, Wakiso, 50% of household income is derived from crop production, with 56% of the population engaged in agriculture ( UBOS, 2016). This implies that while the population engaged in agriculture in Kampala has reduced, in Wakiso this has increased. In this study, we demonstrate that agricultural expansion in urban areas has improved food security but has placed pressure on the wetlands. With projected changes in climate and population increase, wetland encroachment for urban agriculture requires quantitative and reliable agricultural statistics of the productivity of these wetlands. Knowing the exact location and seasonal utilization of these wetlands for agriculture is fundamental for their sustainable use. Periodic information concerning urban agriculture in wetlands can inspire the development of polices that are more inclusive of challenges faced by the urban poor, while at the same time minimize the pressures on urban environments. In addition, protection of these wetlands needs to be intensified to abate negative impacts.

In recent years, monitoring agriculture from space has been effective using remote sensing techniques. Crop characteristics are described in remote sensing using vegetation indices that describe the condition of vegetation in terms of seasonality and land cover change ( Murthy *et al.*, 2007). Vegetation indices are calculated from spectral differences in absorption, transmittance, and reflectance of energy by vegetation in the red and near-infrared regions of the electromagnetic spectrum ( Jensen, 1996). These spectral differences change with the condition of the vegetation in terms of growth or stress, making these indices useful in monitoring agriculture. The normalized difference vegetation index (NDVI), is a commonly used index that is associated with greenness and above ground dry matter by revealing crop photosynthetic activity ( Goward & Huemmrich, 1992; Sarkar & Kafatos, 2004). Agricultural crops exhibit characteristics that are detectable by temporal patterns of NDVI profiles that can be distinguished from other vegetation types through analysis of their respective phenologies ( Guo *et al.*, 2008).

A vegetation sensor aboard the MODIS (moderate resolution imaging spectroradiometer) Terra satellite launched by NASA in 1999 has been used for vegetation monitoring The National Aeronautics and Space Administration (NASA). MODIS NDVI temporal sequences of regularly acquired data (hypertemporal NDVI image data) have been used to monitor drought, vegetation anomalies, vegetation phenology, land cover characteristics, and estimation of crop yields ( Gu *et al.*, 2008; Murthy *et al.*, 2007). Hyper temporal image analysis was first used in the study of monitoring changes in arctic sea-ice by Piwowar *et al*. (1998). A hyper-temporal image analysis involves acquisition of a series of several satellite images of the same area over a period of time. These images are batched together in a self-organising data technique algorithm known as ISODATA clustering. It is followed with a divergence statistical analysis that evaluates signature separabilities, that are used to select the best number of classes present in the NDVI data set, and the correlation between those classes with field data, to develop an informative and user-friendly map ( Nguyen *et al.*, 2012). The objective of this study was to investigate the extent of wetland loss in two Ugandan cities, Kampala and Wakiso, between 1986 and 2016. Secondly, we demonstrate a simple methodology to monitor agriculture on encroached wetlands. The method uses free remote sensing data from Landsat TM and MODIS NDVI 16-day composites at 500-meter spatial resolution to map wetland exploitation, and distinctive crops and crop mixtures in the encroached wetlands.

## Methods

### Study area

**Figure 1.**
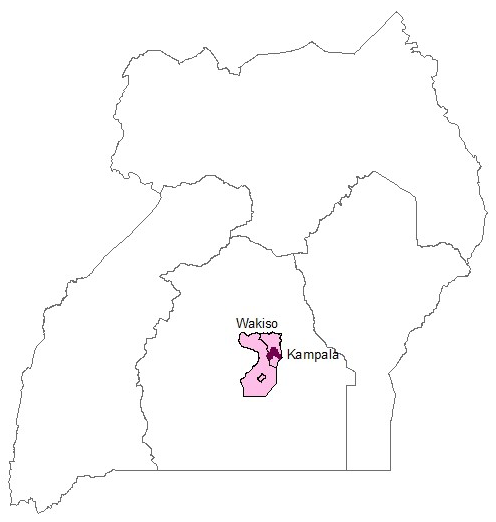
Map of Uganda showing the study area. Kampala the capital city of Uganda (dark colour) and Wakiso district (light colour).

**Figure 2.**
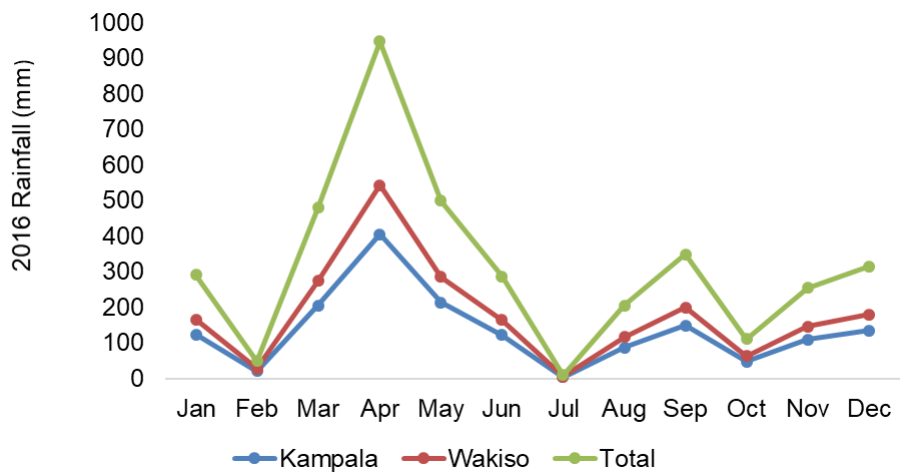
Rainfall pattern of Kampala and Wakiso district during the year 2016 ( UNMA, 2016).

The study area was in Kampala and Wakiso districts with a land area of 176 km ^2^ and 1906.7 km ^2^, respectively. Kampala (0°05′N–0°16′N and 32°30′E–32°38′E) is the capital city of Uganda. Wakiso (0° 24′ 0″ N and 32° 29′ 0″ E), at 59.2% urbanisation level, is the largest urban district and surrounds Kampala in all directions ( Figure 1). The population of Kampala and Wakiso is approximately 1.5 million and 2 million individuals, respectively ( UBOS, 2016). Rainfall data for the year 2016 was obtained from Uganda National Meteorological Authority ( UNMA, 2016) ( Figure 2).

### Landsat TM remote sensing data set

**Figure 3.**
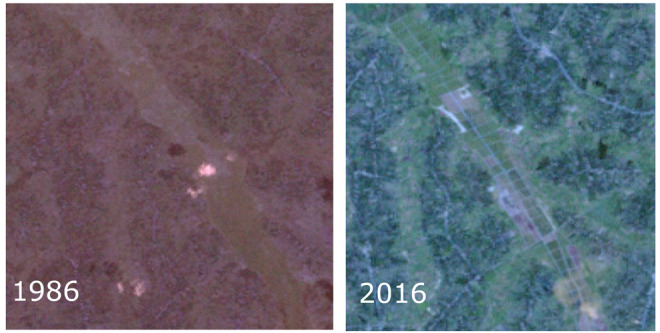
Landsat satellite images showing wetlands converted to agriculture plots in the study area.

Remote sensing data were downloaded from https://earthexplorer.usgs.gov. Landsat image scenes (path 171, row 60, 30m resolution) were acquired for 1986 and 2016. The 1986 scene was from Landsat 5 Thematic mapper (TM), while that of 2016 was from Landsat 7 Enhance Thematic Mapper (ETM). The two images were geo-rectified with topographic maps and with 25 Ground Control Points (GCPs). GCPs are defined as points on the surface of the earth of known location used to geo-reference Landsat Level-1 data. These were identified from https://landsat.usgs.gov/gcp. ERDAS IMAGINE 9.3 software was used for geo-rectification (alternative free software that can perform this task is BEAM, an open-source toolbox and development platform for viewing, analysing and processing of remote sensing raster data: https://earth.esa.int/web/sentinel/user-guides/software-tools/-/article/beam). Labels of classes used in this study included broad categories of land use and land cover, including agriculture, forest, wetlands and agriculture in wetlands ( Figure 3), built up and bare ground:

- Agriculture area: small plots of land or broad tracts of mechanized land areas;
- Forest classification: used training samples from Mabira forest (a natural forest), which was away from the study area on the satellite image, as there were no natural forests within the study area.
- Wetlands: marsh land and seasonal ephemeral areas.

Since a previous data set for the 1986 scene was not available, classification was dependent on the cover types observed and the ground points (109) taken during field work in 2016 (explained below). Agriculture, usually practiced on small plots of land with various crop mixtures with differing crop calendars, increased heterogeneity in agriculture pixels.

The limitation with the 1986 image was the large cloud cover specifically over built-up Kampala area. With this observation, urban areas in the 1986 image were not classified. High resolution images from Google, visual interpretation, ground survey and familiarity of the study area were used to improve the accuracy of the classification. The accuracy of the classification results for 2016 Landsat TM images were assessed using 109 randomly sampled ground truth points, obtained from fieldwork (explained below).

### MODIS surface reflectance 16-day composites

Free remote sensing data was obtained from the MODIS website: https://modis.gsfc.nasa.gov/data/dataprod/mod13.php. The data was visualized through the USGS Global visualization viewer (GloVis), an online search and order tool ( http://glovis.usgs.gov). GloVis was used to select satellite data for the area covering Kampala and Wakiso in Uganda. The MODIS Normalized Difference Vegetation Index (NDVI) collection 5 product MOD13Q1 was used in a hypertemporal optical environment for stratification and crop characterization. MOD13 products estimate ground surface reflectance and are corrected for the effects of atmospheric gases and heavy aerosols. They are masked for water, clouds, and cloud shadows. MOD13 data was accessed as 16-day composites at 500-meter spatial resolution. Each pixel encompasses the best observation values within every 16-day period. Each image includes a Blue, Red and Near Infrared reflectance band. These bands are centred at 469, 645 and 858-nanometers, respectively, from which the NDVI (equation 1) was calculated. MODIS 16-day composite images from April-September 2016 were used for deriving 13 NDVI images. The specific dates of the satellite: 2 ^nd^ and 18 ^th^ February, 5 ^th^ March, 6 ^th^ and 22 ^nd^ April, 8 ^th^ and 24 ^th^ May, 9 ^th^ June, 27 ^th^ July, 12 ^th^ and 28 ^th^ August, 13 ^th^ and 29 ^th^ September 2016. The choice of the dates was based on the availability of the satellite but followed the bimodal rainy season that starts from March to June and from August to November.

NDVI = ( *NIR* – *Red*)/( *NIR* + *Red*)             (1)

### MODIS-based stratification and characterization of urban agriculture in wetlands

Using ERDAS IMAGINE 9.3 software, the 13 NDVI images were stacked one over the other in chronological order of the dates (alternative free software that can perform this task is BEAM).

The stacked images were then batched to create one image that was subset to create a sub-area of Wakiso and Kampala. MODIS unsupervised classification was carried out with ERDAS IMAGINE. The unsupervised classification was then performed using an ISODATA batch run calculated using the divergence distance measure to assess the clustering signature separability ( Asilo *et al.*, 2014). A total of 50 classes from the stratified random sampling were derived. To generate the digital NDVI numbers (DN), linear stretching was applied. The minimum NDVI value, -1, was assigned 0 while the maximum NDVI value, 1, was assigned values from 1 to 255. The extraction of 50 classes provided an ideal stratification for the NDVI time series. The NDVI ISODATA image was polygonised in ERDAS IMAGINE software and plotted as a map in ArcGIS software version 10.3 (alternative software that can perform this task is QGIS, a free and open source software: https://www.qgis.org/en/site/).

### Field work

All the 50 class polygons were given a unique identifying colour and located on a boundary map of both Wakiso and Kampala. The data was input on ArcPad 10.3 on a Trimble GPS system that was used in the field (in addition to QGIS, Google maps App on an android phone can also perform this task). In the period between April and December 2016, the sites on the maps were visited through a mobile GIS approach, which consisted of a sensitive hand held Gramin Global Positioning System (GPS) and Trimble GPS system running ArcPad to locate the ground truth points. Due to rough terrain and inaccessibility of some wetland areas, sample points that were near roads were selected. The ground truth points were saved in ArcPad for later use. For all 50 classes, 5 sites of each class were visited giving a total of 250 sites in Kampala and Wakiso. The sampling frame consisted of 20 clusters of five sampling units. Each sampling unit within a cluster had a 15-meter radius of sampling area whose centres were 120 meters apart within each cluster. The data collected included 250 ground truth points (XY coordinates), cover percentage of vertical vegetation, dominant species, land cover and land use. These gave an indication of types of crops growing on the site and the associated wetland encroachment. Data collected from field work was checked for completeness and was organized using Excel for analysis.

### Accuracy assessment

Accuracy assessment of the MODIS-derived urban agriculture was based on the kappa coefficient and confusion matrix assessing classified pixels with reference to ground-truth points. The urban agriculture maps generated from MODIS was evaluated using 109 randomly sampled ground truth points, obtained from the fieldwork explained above. To identify homogenous classes based on NDVI characteristics, similarity measures between the 50 classes were generated from hierarchical cluster analysis using Pearson’s correlation as the proximity procedure. The clustering analysis was conducted in SPSS version 20.0 ( SPSS, 2011).

## Results

### Rainfall

Usually Kampala and Wakiso district location around the Lake Victoria basin is characterized by two rainy seasons. However, 2016 had one prominent rainy season that started in February, peaked in April (940 mm) and gradually dropped in July, which is a usual rain pattern in the first season of the study area. In the second half of the year, the rainfall pattern was more erratic with the amount of rainfall barely attaining 400 mm between September and December of 2016. This prominently dry season resulted in one of the worst droughts Uganda has faced in recent years.

### Current state of urban wetlands in Uganda

**Figure 4.**
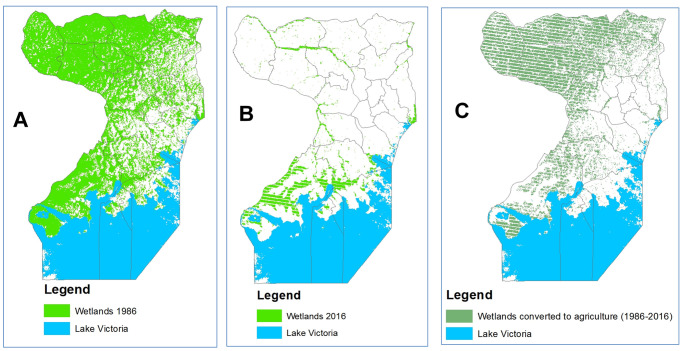
Landsat TM images showing 30-year land cover, revealing wetland encroachment by agriculture in Wakiso and Kampala. Wetlands, 1986 ( **A**), wetlands, 2016 ( **B**) and wetlands converted to agriculture between 1986 and 2016 ( **C**).

**Figure 5.**
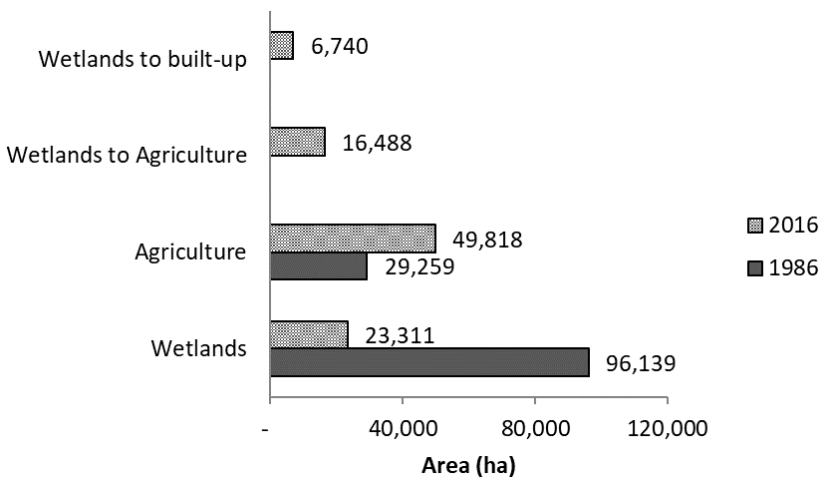
The 30-year change in area of wetlands in the study area and the state of encroachment for agriculture and built-up areas in Wakiso and Kampala.

The overall classification accuracy and Kappa coefficient for 1986 and 2016 land cover maps of the Wakiso-Kampala study area was 83.1% and 0.87, and 87% and 0.85, respectively. We found that over the 30-year period, 72,828 ha of the Wakiso-Kampala wetlands have been lost ( Figure 4 and Figure 5). Agriculture on the other hand doubled in cultivation area. Of the new cultivation area, 16,488 ha have been reclaimed from wetlands. The overall accuracy and Kappa coefficient for the MODIS based stratification Wakiso-Kampala study area for crop agriculture in the wetlands was 92% and 0.93, respectively. Our results showed that all crop agriculture segregated in Kampala using hyper temporal remote sensing was in the wetlands, while 73% of the crop agriculture segregated in Wakiso was in the wetlands.

### Characterization of crop agriculture in wetlands

**Figure 6.**
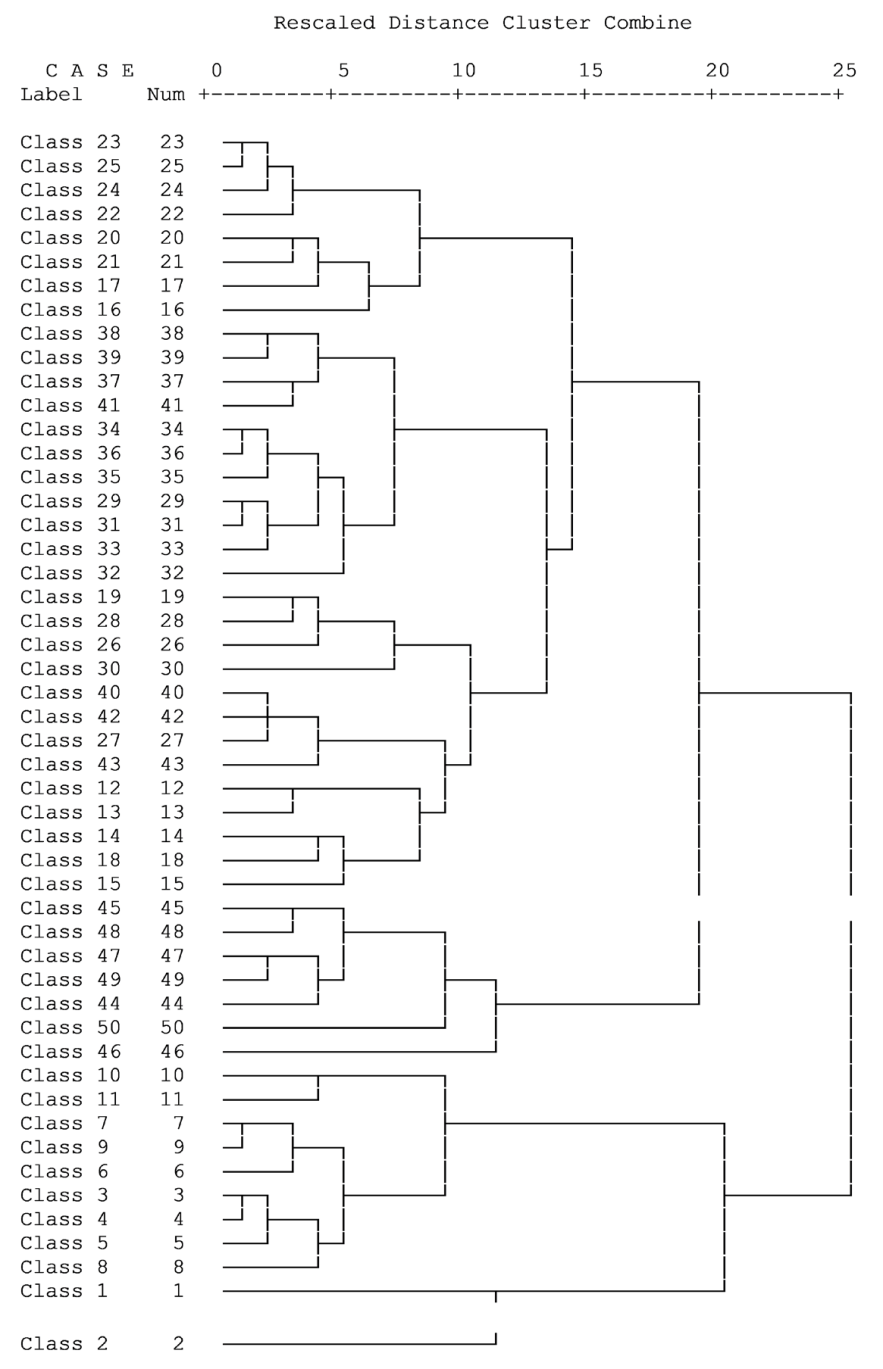
Dendogram showing grouping of 50 NDVI classes segregated by hierarchical cluster analysis.

**Figure 7.**
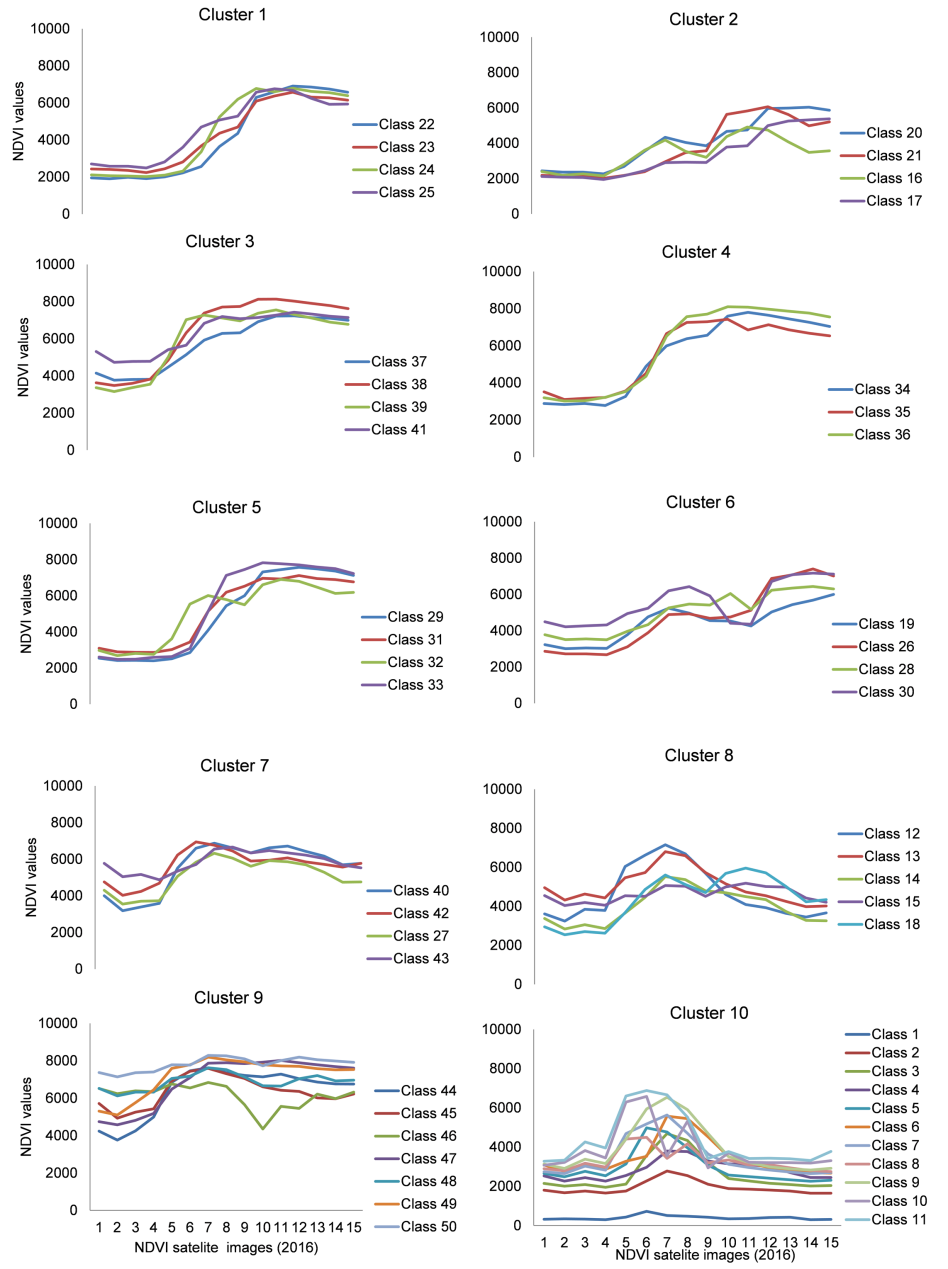
Respective profile curves of NDVI plotted from clusters produced by the dendogram in Figure 6. Numbers 1 to 15 in the legend refer to the chronology of dates of the satellites taken every 16 days in the two 2016 growing seasons of Kampala and Wakiso.

Temporal signatures of the 50 NDVI classes were obtained. These were plotted in a spread sheet to give profile curves of NDVI DN values for each 16-day composite from April to September 2016 ( *Extended data*). Hierarchical cluster analysis yielded a dendogram ( Figure 6) that segregated the 50 NDVI classes into 10 clusters ( Figure 7). Five of the clusters (cluster 1–5) showed sigmoid curves of NDVI values as the year progressed. These five clusters appeared similar but differed in values at the beginning of the year and at the levelling off points. The NDVI profiles in cluster 1 (classes 22–25), started off at 2000–3000 values and levelled off between 7000–6000 values, while the profiles in cluster 2 (classes 16, 17, 21 & 20), also started off at 2000–3000 values but levelled off between 6000–5000 values but were more erratic in shape. The NDVI profiles in cluster 3 (classes 37–39 & 41), started off at 3500–5500 values, levelled off below 8000 while the NDVI profiles in cluster 4 (classes 34–36) started off at 5000 values, also levelled off below 8000 values but had a smoother sigmoid growth than cluster 3. Cluster 5 (classes 29, 31–33) was similar to cluster 3 but differed by starting off at 3000 values and had steeper growth than cluster 3. Clusters 6, 7 and 8 were similar but differed in their exhibition of prominent peaks during the course of the year. The NDVI profiles in cluster 6 (classes 19, 26, 28 & 30) started off at 3000 values but peaked in early May just above 6000 values, dipped deeply in early July (just above 4000 values) and peaked again in September (about 7000 values). The NDVI profiles in cluster 7 (classes 27, 40, 42 & 43) started off between 4000–6000 values but peaked in early May just above 6000 values, slightly dipped in early June (just below 6000 values) and peaked again in late July (about 6500 values). The NDVI profiles in cluster 8 (classes 12–15, & 18), on the other hand, started off between 2500–5000 values, prominently peaked in early May (just above 6000 values), strongly dropped in early July (just below 6000 values) and conspicuously peaked again in mid-August but at lower values (below 6000) than they did in May.

The NDVI profiles of cluster 9 (classes 44–50) were flatter throughout the year except the NDVI profile of class 46 that prominently dipped in early July but rose gradually during the last quarter of the year. The NDVI profiles of cluster 10 (classes 1–10) were conspicuously different from all the other nine clusters in that the first quarter of the year started off with flat profiles ranging between 1800–3800 values (except class 1). However, in late March, the profiles rose drastically to just above 6000 values and peaked in late April and then gradually dropped to early July and then remained flattened out for the rest of the year.

**Figure 8.**
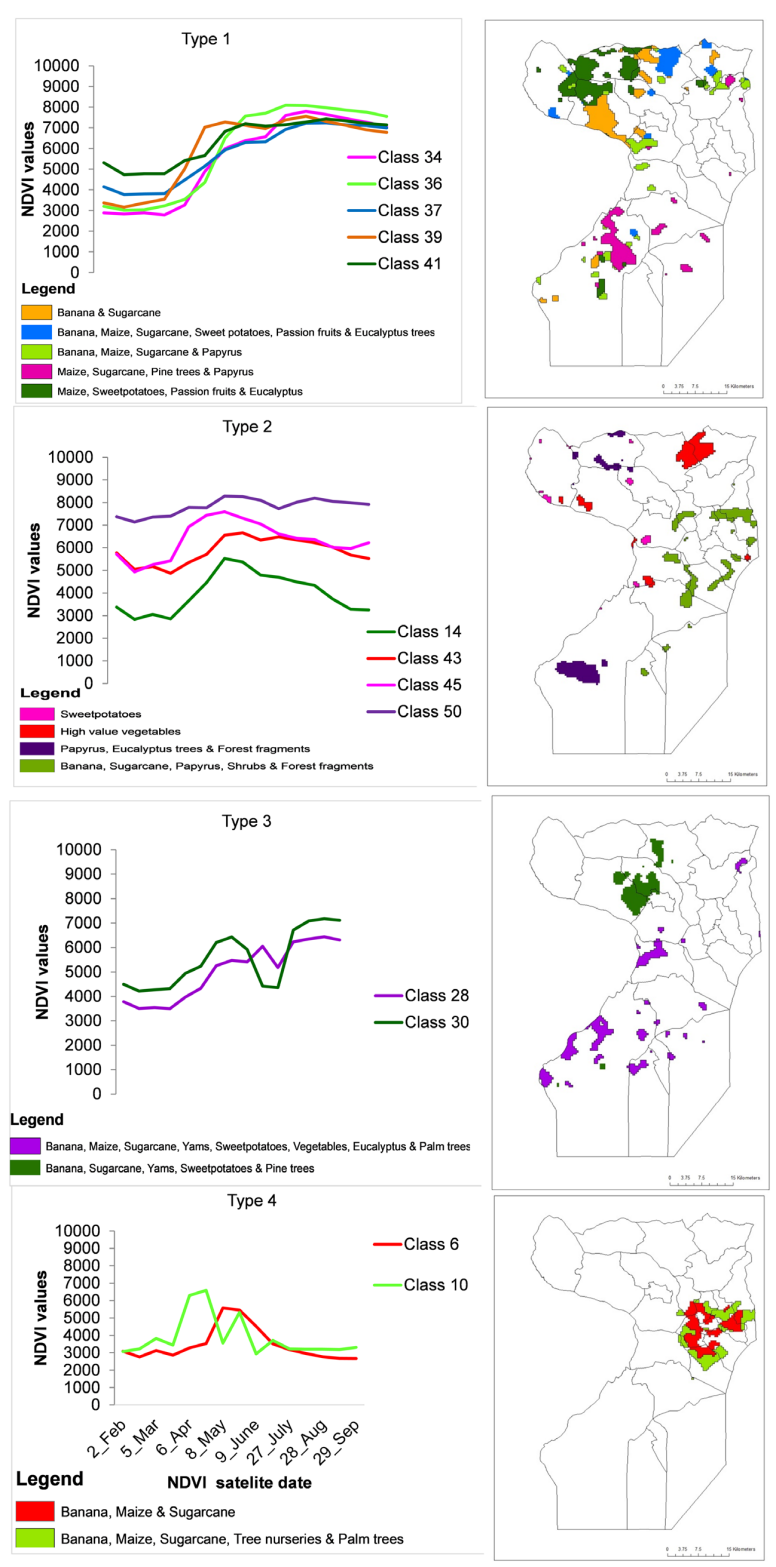
Types of crops and crop mixtures in Uganda’s urban wetlands. On the right is a Wakiso-Kampala map showing the land area corresponding to a respective crops and crop mixtures in a similar colour.

Ground observations found that of 50 classes represented in the 10 clusters, 13 of them represented agriculture in the wetlands. The wetland classes included classes 6, 10, 14, 28, 34, 36, 37, 39, 41, 30, 43, 45 and 50. These were represented in all clusters except clusters 1, 2 and 5. The major crops grown in these urban wetlands in order of frequency were banana (20%), sugarcane (22%), maize (17%), *Eucalyptus* (12%) and sweet potatoes (10%), while ornamental nurseries, pine trees, vegetables and passion fruits were each at 5%. Using visual interpretation, the 13 classes in the wetlands were graphed using similarity of the shape NDVI profiles, which yielded 4 types of phenology types. Type 1 included NDVI classes 34, 36, 37, 39 and 41, while Type 2 included 14, 43, 45 and 50. Type 3 included classes 28 and 30 while Type 4 included classes 6 and 10. The crops and crop mixtures that each of these classes represent are shown in maps in Figure 8.

## Discussion

Our results clearly show that wetlands in Uganda’s urban areas have been the prime target for agricultural expansion in the last 30 years. The results reveal that 76% of wetlands in the Wakiso-Kampala study area have been lost. Of the lost wetlands, 23% have been converted to agricultural cultivation area. Using The MODIS-based classification of urban agriculture we showed that monitoring agriculture in these wetlands using ISODATA analysis, is a worthwhile approach to manage, monitor and control the elusiveness of wetland encroachment. The normalized difference vegetation index (NDVI) from MODIS revealed crop characteristics that were detectable by temporal patterns of NDVI profiles distinguishing crops and crop mixtures through analysis of their respective phenologies. The NDVI profiles were sensitive to the rainfall pattern, which in 2016 consisted of one rainfall season and an erratic second season. This anomaly was rather unsurprising as in recent years Uganda has shown high vulnerability to rainfall variability and climate change ( MoWE, 2010). Many studies have observed and validated a linear relationship between NDVI and precipitation, an association that is highly sensitive to climatic fluctuations ( Nicholson *et al.*, 1990; Nicholson & Farrar, 1994; Wang *et al.*, 2003). Our results show that at the peak of the rainy season in early April, the NDVI profiles started rising irrespective of the category of the NDVI classes, confirming the linearity of the NDVI-precipitation relationship, mentioned above. We also observed that some NDVI profiles in wetland classes levelled off during the month of July and plateaued through the rest of the year. This implied that the crops grown in the wetlands responded strongly to the rainfall season but also remained thriving during the prolonged drought. Although most of the country suffered an acute food insecurity situation, which saw Uganda loose her food secure status ( OPM, 2017), crops in these urban wetlands exhibited resilience, owing to the moisture retained in these wetter ecosystems; wetlands are linked to accumulation of fertile sediment during floods and long periods of water retention ( Dixon & Wood, 2003).

The 13 classes of NDVI profiles identified in the wetlands seemed to be determined by the type of agriculture practiced in these wet ecosystems. The vegetation in Type 1 was prominent in western Wakiso in the sub counties of Kakiri, Masuliita, Gombe and Kasanje whose wetlands are made of papyrus flora, which is the natural wetland vegetation. The NDVI profiles in this type had sigmoid curves that rose in early April at the peak of the rainy season, depicting a crop phenology that followed the rains or one that responded strongly to high moisture. This assertion was confirmed by ground data that observed that Phenology Type 1, was dominated by perennial crops (bananas: *Musa* spp; sugarcane: *Saccharum officinarum*) *,* seasonal crops (specifically maize: *Zea mays*), fruit farming (specifically passion fruit: *Passiflora edulis*) and silviculture (pine trees: *Pinus* spp. and *Eucalyptus* spp.). The vegetation in Type 2 was prominent in Wakiso in the sub counties of Busukuma, Kiira, Sbagabo-Makindye and Kasanje which are more peri-urban sub counties with a large influence of Kampala city in terms of urban markets. The profiles in Type 2 were flatter curves depicting a continuous crop phenology that can either represent uninterrupted crop growth or perennial crops. Field observations found that Type 2 was dominated by high value vegetables (radish: *Raphanus* spp.; broccoli: *Brassica oleracea;* lettuce: *Lactuca sativa*; sweet potatoes: *Ipomoea batatas)*, which are constantly cropped, and perennial crops such as banana and sugarcane. This finding is indicative of the advantage of the constant moisture supply provided by these wetlands. The vegetation in Type 3, was prominent in Wakiso in the sub counties of Kasanje, Wakiso Town council, Kakiri and Gombe. The profiles in Type 3 rose sharply in early April, depicting vegetation response to rainfall and fell in early July portraying harvest, but rose sharply again in late July, representing the commencement of a second cropping season. Field observations found that Type 3 was a mixture of crops found in both Type 1 and Type 2 that were perennial, annual, silviculture and horticulture, still indicating the extent of which urban wetland ecosystems offer a substantial supply of moisture to carry two cropping seasons. Type 4 was prominently in the wetlands of the Greater Kampala metropolitan division of Nakawa, Rubaga, Makindye and the Central city area. The most prominent crops were banana, maize, sugarcane and tree nurseries.

Some studies have shown that urban wetlands in Uganda contribute approximately US $432 per year to local communities practicing subsistence agriculture ( Turyahabwe *et al.*, 2013). Moreover, the type of crops grown in the urban wetlands are important Ugandan staple crops that have a high economic value in urban markets but are also a reflection of urban nutritional combinations. It is not surprising that bananas dominated urban wetland agriculture, as these are the country’s staple crop. Sugarcane and fresh roasted maize are enjoyed by urban dwellers as snacks and are sold along roadsides. In other countries in sub-Saharan Africa, an increasing population in combination with efforts to increase food security has intensified pressure to expand agriculture in wetlands. For instance, in many parts of eastern and central Africa, it has been observed that up to three crops per year can be grown in wetlands significantly contributing to food security. For example, in Tanzania, the Kilombero wetland was found to contribute up to 98% of food intake for all households surveyed irrespective of the socioeconomic status ( Rebelo *et al.*, 2010).

It has been suggested that wetlands can be converted to include intensification of a specific wetland strategy, such as the complete reclamation or commercial agriculture or industrial development, which are considered to be more economically viable ( Hollis, 1990). Conversely, it has been a matter of debate whether quantifying the economic value of wetlands in Africa undervalues their importance for their future utilization ( Schuyt, 2005a; Seyam *et al.*, 2001). For example, already, the essential role that wetlands play in regulating the flow of water into the Lake Victoria has been lost ( Olindo, 1992). A couple of decades ago, agriculture was responsible for 80% and 75% riverine phosphorus and nitrogen entering Lake Victoria ( Odada *et al.*, 2004; Scheren *et al.*, 2000). Papyrus wetlands play a significant role of filtration and protection of the lake from eutrophication acting as sediment traps and buffer discharges ( Ryken *et al.*, 2015). Whereas short term impacts are already visible, studies on longer term impacts of such massive wetland encroachment at both local and regional scales are limited. The danger is that the current wetland exploitation for food security may be a trade-off between the provision of food in the short-term and the loss of important ecosystems services in the long-term.

### Policy implications

The significant policy implications from this study depict that the Kampala-Wakiso wetlands have been disappearing at a rate of 2500 ha annually for the last 30 years, implying that at this rate, there will be no wetlands left by 2029. In this respect, it means that ecosystem services of these urban wetlands have been lost. It could be that the main challenge in implementation of policies that effectively protect these wetlands is that legislative and policy provisions have lagged behind growing scientific knowledge and understanding. Matching policy to cutting edge science can minimize and mitigate the impacts on ecosystems resulting from over exploitation ( MacKay, 2006). Government environmental protection bodies have access to widely applied and tested methods of assessing wetland encroachment at larger scales, such as remote sensing data ( WETLANDS-ATLAS, 2016). These institutions can integrate regional and local databases to identify potentially vulnerable wetland dependent ecosystems. Scientists on the other hand can develop the scientific knowledge on understanding wetland dependent ecosystems at both local and regional scales. When these two levels of understanding are merged, this information can be useful in implementation and strengthening of already existent but poor policies. For example modelling scenarios of threatened and vulnerable ecosystems to policies, can be used to predict the future of wetland encroachment as evidence based data to strengthen weak policies ( Odgaarda *et al.*, 2017). In addition, the ability to address multiple approaches that identify the various ecosystem services provided by wetland ecosystems through rapid assessment of wetland ecosystem services is required. These can provide an output of a range of ecosystem services through a rapid and comprehensive overview of the various benefits provided by wetlands ( Everard & McInnes, 2013; McInnes & Everard, 2017). The Rapid Assessment of Wetland Ecosystem Services (RAWES) approach interactively involves all stakeholders and equips wetland managers to address data constraints in relation to the magnitude and extent of beneficiaries. The benefits are linked through three scales: local benefits (at household and individual level), regional benefits (at wider catchment levels), and global benefits (those beyond national boundaries) ( McInnes & Everard, 2017). Such an approach could sufficiently increase the ability to recognize the importance of ecosystem services, monetary valuation and multiplicity of social economic benefits of these urban wetland.

The second policy implication from this study indicates that a large population of urban dwellers are utilizing these wetlands for survival and therefore confirming the observation that poverty eradication policies are in conflict with wetland conservation policies. This study could not ascertain the fraction of commercial from subsistence farming that was provided by these urban wetlands as it was not easy to identify owners of the crops in the wetlands. It is possible that urban dwellers farming in wetlands are aware that it could be illegal, but do not understand the framework of the impropriety; they take care of crops very early in the morning and then have other occupations during the day. Policy regulators on the other hand observe growing crops but cannot identify the owners or whether the agricultural practice used is suitable for wetlands. This indicates that there lacks sensitization of simple but precise indicators of what wetland encroachment for agriculture is to lay persons. In addition, government protection dialogue with relevant stakeholders may be rather high handed, with reports of destroying food crops grown in wetlands or forceful evictions) ( DISPATCH, 2019; Monitor, 2017). The tendency to emphasize discipline-bound legislations could easily have demoralized citizens from recognizing potential economic and ecosystem services of these urban wetlands. This has in turn undermined conservation of biodiversity and weakened protection laws. At the same time wetlands are seen as an easy option for the construction of infrastructure. For instance, in recent years, to avoid compensation to evacuated urban settlements on road reserves, major roads have been constructed in the middle of papyrus wetlands. In return, flood events have increased in adjacent areas that are hazardous to the most vulnerable urban poor ( African Development Fund, 2008; Isunju *et al.*, 2016b). Already, in Wakiso district, wetland encroachment for settlement and agriculture has changed the local area climate in terms of increasing drought, reductions in rainfall seasons and increasing day and night temperatures ( WDLG, 2017). This may have far-reaching consequences to local communities dependent on these wetlands but have significantly contributed to the environmental crisis at the Lake Victoria basin.

### Policy recommendations

We recommend that the Government of Uganda needs to promote wetland reclamation programs so as to restore and reconstruct lost and fragmented wetlands. Secondly, ministries mandated to food security and poverty eradication need to convene with ministries regulating wetlands to merge these conflicting policies. Thirdly, there is need for the development of polices that are inclusive of challenges faced by the urban poor while at the same time minimize the pressures on urban environments.

## Conclusion

The level of wetland degradation revealed by this study shows that protection of urban wetlands has been relatively low pointing to poor policy implementation over the years. Despite the existence of seven policies protecting wetlands, enforcement and compliance systems have not been suited for the dynamicity of urban growth. This study has demonstrated that despite environmental data being scarce and heterogeneous landscapes in Africa being difficult to map, wetland regulators in Uganda can utilize free remote sensing data to monitor wetlands. In the present study, the MODIS Collection 5 land cover datasets of high resolution (500 m) processed with the ISODATA clustering algorithm was a useful choice of remote sensing data for monitoring wetland encroachment by crop agriculture. The necessity of collecting training datasets of individual crops grown in the wetlands during field work was key to ascertain the results of ISODATA clustering of NDVI profiles. This greatly improved the accuracy of our maps. The NDVI time series from ISODATA clustering algorithm have been shown to capture crop phenologies (crop calendars) and thus represents a good relationship with the cropped areas.

The dynamic situation of these urban wetlands requires informed understanding of the ecological and socio-economic benefits that they provide. There is a need to recognize the longer-term degradation threats and more spatially extensive impacts of these changes. This calls for coordinated adaptation strategies between scientists, policy makers and urban dwellers for equitable utilization of wetlands without compromising their ecosystem services and economic benefits.

## Data availability

### Underlying data

Remote sensing data: https://earthexplorer.usgs.gov

MODIS remote sensing data: https://modis.gsfc.nasa.gov/data/dataprod/mod13.php

1. Satellite imagery from LAND SAT (1986 and 2016):
  a. Pangaea: Detecting level of wetland encroachment for urban agriculture in Uganda using hyper-temporal remote sensing - *Land_stat_1996*, https://doi.pangaea.de/10.1594/PANGAEA.913482 ( Kabiri *et al.*, 2020a).
  b. Pangaea: Detecting level of wetland encroachment for urban agriculture in Uganda using hyper-temporal remote sensing - *ground truth points of wakla,* https://doi.pangaea.de/10.1594/PANGAEA.913486 ( Kabiri *et al.*, 2020b).
  c. Pangaea: Detecting level of wetland encroachment for urban agriculture in Uganda using hyper-temporal remote sensing - *classifiedlandstat2016*, https://doi.pangaea.de/10.1594/PANGAEA.913480 ( Kabiri *et al.*, 2020c).
2. Satellite imagery from SPOT NDVI (monthly 2015–2016):
  a. Pangaea: Detecting level of wetland encroachment for urban agriculture in Uganda using hyper-temporal remote sensing - *Gapmark2016,* https://doi.pangaea.de/10.1594/PANGAEA.913481 ( Kabiri *et al.*, 2020d).
3. ISODATA clustering of NDVI profiles for 50 classes and wetland class types:
  a. Pangaea: Detecting level of wetland encroachment for urban agriculture in Uganda using hyper-temporal remote sensing, https://doi.pangaea.de/10.1594/PANGAEA.915587 ( Kabiri *et al.*, 2020e).
4. GIS shape files for wetland encroachment for the last 30 years:
  a. Pangaea: Detecting level of wetland encroachment for urban agriculture in Uganda using hyper-temporal remote sensing, https://doi.pangaea.de/10.1594/PANGAEA.915586 ( Kabiri *et al.*, 2020f).

Data are available under the terms of the Creative Commons Attribution 4.0 International license (CC-BY 4.0).
