# Detecting wetland encroachment and urban agriculture land classification in Uganda using hyper-temporal remote sensing

**DOI:** 10.12688/aasopenres.13040.2

**Published:** 2022-02-16

**Authors:** Stella Kabiri, Molly Allen, Juduth Toma Okuonzia, Beatrice Akello, Rebecca Ssabaganzi, Drake Mubiru

**Affiliations:** 1 Mukono Zonal Agricultural Research Institute (MUZARDI), National Agricultural Research Organisation, Mukono, P.O.Box. 164, Mukono, Uganda; 2 National Livestock Resource Research Institute (NARLRRI), National Agricultural Research Organization, Kampala, P. O. Box 5706, Uganda; 3 Faculty of Natural Resources and Environmental Sciences, Busitema University, Tororo, P.O.236, Uganda; 4 Wakiso District Local Government, Department of Natural Resources, Wakiso, Uganda; 5 National Agricultural Research Laboratories (NARL) of the National Agricultural research Organization, Kampala, P.O.Box 7065, Uganda

**Keywords:** Environmental degradation, Papyrus wetlands, Lake Victoria, Urban growth, Sustainability

## Abstract

**Background: **Urbanization is an important indicator of economic growth and social change but is associated with environmental degradation, which threatens the sustainable growth of African cities. One of the most vulnerable ecosystems in urban areas are wetlands. In Uganda, wetlands cover an area of 11% of the country’s land area. Half of the wetland areas in Ugandan cities have been converted to industrial and residential areas, and urban agriculture. There is limited information on the extent of wetland conversion or utilization for urban agriculture. The objective of this study was to investigate the extent of wetlands lost in two Ugandan cities, Wakiso and Kampala, in the last 30 years. Secondly, we extracted crop agriculture in the wetlands of Kampala and Wakiso from hyper-temporal satellite image analysis in an attempt to produce a spatial detail of wetland encroachment maps of urban agriculture using a reproducible mapmaking method.

**Methods: **Using a field survey and free remote sensing data from Landsat TM 1986 and Landsat ETM 2016 we classified the rate of wetland loss and encroachment between the years 1986 and 2016. We used MODIS NDVI 16-day composites at a 500-meter spatial resolution to broaden the analysis to distinguish distinctive crops and crop mixtures in the encroached wetlands for urban agriculture using the ISODATA clustering algorithm.

**Results:** Over 30 years, 72,828 ha (73%) of the Wakiso-Kampala wetlands have been lost meanwhile agriculture areas have doubled. Of this 16,488 ha (23%) were converted from wetlands. All cultivated agriculture in Kampala was in the wetlands while in Wakiso, 73% of crop agriculture was in the wetlands. The major crops grown in these urban wetlands were banana (20%), sugarcane (22%), maize (17%), *Eucalyptus* trees (12%), sweet potatoes (10%), while ornamental nurseries, pine trees, vegetables, and passion fruits were each at 5%.

## Introduction

While global urbanization is stipulated to increase to 67% by 2050, Africa’s urban population is predicted to triple ( UN-HABITAT, 2012). Although urbanization is an important indicator of economic growth and social change, this fast growth is associated with environmental degradation, which threatens sustainable growth of African cities. One of the most vulnerable ecosystems in urban areas are wetlands. Wetlands of the world cover 9% of the global land area ( Zedler & Kercher, 2005). Human induced activities driven by population pressure, expansion of agricultural land area, land degradation and poor policies have led to the loss of at least 50% of the global wetland land area ( Chapman *et al.*, 2001; Hartter & Southworth, 2009; IUCN, 1996). As a result ecosystem services performed by wetlands, such as water quality improvement, flood abatement, carbon sequestration, biodiversity ecological units of wild life and medicinal plants, have been reduced ( Dugan, 1993; Joosten, 2009; Saunders *et al.*, 2012; Schuyt 2005b; Zedler & Kercher, 2005).

In Uganda, wetlands cover an area of 11% of the country’s land area, with seasonal wetlands covering 7.7%, while permanent wetlands and swamp forests cover 3.4% and 0.1%, respectively (e.g. WETLANDS-ATLAS, 2016). A recent study in Kampala by Abebe (2013) showed that 658 hectares of permanent wetlands in Kampala, Uganda’s capital, had been converted to built-up areas between 1989 and 2010. However, there exists limited information on the extent of wetland conversion or utilization for urban agriculture. There is evidence that former rural farmers who migrate to urban areas transfer rural livelihood strategies by engaging in urban agriculture, which is most often in the wetlands ( Isunju *et al.*, 2016a). Wetland encroachment has increasingly become hazardous to the most vulnerable urban poor whose livelihoods depend on their immediate environment ( African Development Fund, 2008; Isunju *et al.*, 2016b). In spite of the hazards, food security of livelihoods living around wetlands is supported by abundant soil moisture and fertile sediments used for crop farming almost throughout the year ( Turyahabwe *et al.*, 2013). Despite the importance and value of these services for many people, wetlands are also amongst the most threatened ecosystems globally, especially from the effects of agriculture ( Falkenmark *et al.*, 2007; Finlayson, 2010). In sub-Saharan Africa, policy makers face a dilemma of policy regulations with wetlands, as they support the livelihoods of many poor people through the provision of numerous ecosystem services, including food ( Bikangaga *et al.*, 2007).

Uganda has seven policies that emphasize optimization of sustainable benefits of wetlands, while conserving the environment and biodiversity. These policies include The National Policy for the Conservation and Management of Wetlands of 1995, the National Environment Act of 1995, the Land Act of 1997, the Local Government Act of 1997, the Environment Impact Assessment Regulations of 1998, the Wetland Regulations of 2000, and the Constitution of 2010 ( WETLANDS-ATLAS, 2016). These policies emphasise protection of wetlands and forbid any form of wetland reclamation ( Isunju *et al.*, 2016a). They are enforced by the National Environmental Management Authority (NEMA), the Kampala Capital City Authority (KCCA) and the Ministry of Water and Environment, who call for eviction of wetland encroachers ( Isunju *et al.*, 2016a; MWE, 2001). Nevertheless, despite these policy interventions, half of the wetland areas in Ugandan cities have been converted to industry and residential areas, and crop land ( MWE, 2014; UBOS, 2009). The presidential initiative of Operation Wealth Creation ( OWC, 2017) and Uganda’s Vision 2040 policies ( NDPII, 2015) include increasing the ability of the poor to raise incomes and improve the quality of life of the poor. Wetlands in Ugandan cities are a key source of livelihood for the urban poor and yet over exploitation can lead to land degradation and risk of food shortages. This implies that there lies a dilemma in implementing these wetland conservation policies in the same framework as Operation Wealth Creation ( OWC, 2017), Sustainable Development Goal 11 (Sustainable cities and communities) and Uganda’s Vision 2040 policy ( NDPII, 2015), in regards to urban areas.

Twenty years ago, 35% of Kampala households engaged in agriculture within the city ( Maxwell, 1995). Agriculture land in Kampala comprised of a total of 11, 942 hectares which was 56.1% of the total land area of the city ( Maxwell, 1995). A recent study observed that currently the population of Kampala engaged in agriculture has dropped to 5.1%, and yet 38% of household income was from crop production ( UBOS, 2016). In another urban district, Wakiso, 50% of household income is derived from crop production, with 56% of the population engaged in agriculture ( UBOS, 2016). This implies that while the population engaged in agriculture in Kampala has reduced, in Wakiso this has increased. The economic value and social-economic benefits such as crop agriculture in the wetlands of urban Uganda have been published widely (e.g. in Emerton *et al*., 1998; Kakuru *et al*., 2013; Turyahabwe *et al*., 2013; UNDP/NEMA/UNEP, 2009). However, none provides the spatial detail of these economic benefits about wetland encroachment using a reproducible mapmaking method. In our research, we extracted crop agriculture in the wetlands of Kampala and Wakiso from hyper-temporal satellite image analysis in an attempt to produce detailed and reproducible wetland encroachment maps of urban agriculture. With projected changes in climate and population increase, wetland encroachment for urban agriculture requires quantitative and reliable agricultural statistics of the productivity of these wetlands. Knowing the exact location and seasonal utilization of these wetlands for agriculture is fundamental for their sustainable use. Periodic information concerning urban agriculture in wetlands can inspire the development of policies that are more inclusive of challenges faced by the urban poor, while at the same time minimizing the pressures on urban environments. In addition, the protection of these wetlands needs to be intensified to abate negative impacts.

In recent years, monitoring agriculture from space has been effective using remote sensing techniques. Crop characteristics are described in remote sensing using vegetation indices that define the condition of vegetation in terms of seasonality and land cover change ( Murthy *et al*., 2007). Vegetation indices are calculated from spectral differences in absorption, transmittance, and reflectance of energy by vegetation in the red and near-infrared regions of the electromagnetic spectrum ( Jensen, 1996). These spectral differences change with the condition of the vegetation in terms of growth or stress, making these indices useful in monitoring agriculture. The normalized difference vegetation index (NDVI), is a commonly used index that is associated with greenness and above-ground dry matter by revealing crop photosynthetic activity ( Goward & Huemmrich, 1992; Sarkar & Kafatos, 2004). Crops exhibit characteristics that are detectable by temporal patterns of NDVI profiles that can be distinguished from other vegetation types through analysis of their respective vegetation phenologies ( Guo *et al*., 2008). Vegetation phenology refers to the patterns and characteristics of plants that transform with the seasons or the study of the timing of recurring seasonal biological events of terrestrial ecosystems ( Schwartz, 2003). A vegetation sensor aboard the MODIS (moderate resolution imaging spectroradiometer) Terra satellite launched by NASA in 1999 has been used for vegetation monitoring (NASA). The MODIS sensor measures the leaf area index (LAI) of satellite reflectance information ( Kang *et al*., 2003). Regularly acquired hyper-temporal NDVI image data have been used to monitor vegetation phenology, drought, vegetation anomalies, land cover characteristics, and estimation of crop yields ( Gu *et al*., 2008; Murthy *et al*., 2007). Hyper-temporal image analysis was first used in the study of monitoring changes in arctic sea-ice by Piwowar *et al.* (1998). Hyper-temporal image analysis involves the acquisition of a series of several satellite images of the same area over a period of time. These images are batched together in a self-organizing data technique algorithm known as ISODATA clustering ( ERDAS, 2005; Girma *et al*., 2016). It is followed by a divergence statistical analysis that evaluates signature separabilities, that are used to select the best number of classes present in the NDVI data set, and the correlation between those classes with field data, to develop an informative and user-friendly map ( Nguyen *et al*., 2012). The objective of this study was to investigate the extent of wetland loss in two Ugandan cities, Kampala and Wakiso, in the last 30 years. Secondly, we extracted crop agriculture in the wetlands of Kampala and Wakiso from hyper-temporal satellite image analysis in an attempt to produce a spatial detail of wetland encroachment maps of urban agriculture using a reproducible mapmaking method. We used free remote sensing data from Landsat TM and MODIS NDVI 16-day composites at a 500-meter spatial resolution to map wetland exploitation, and distinctive crops and crop mixtures in the encroached wetlands.

## Methods

### Study area

**Figure 1.**
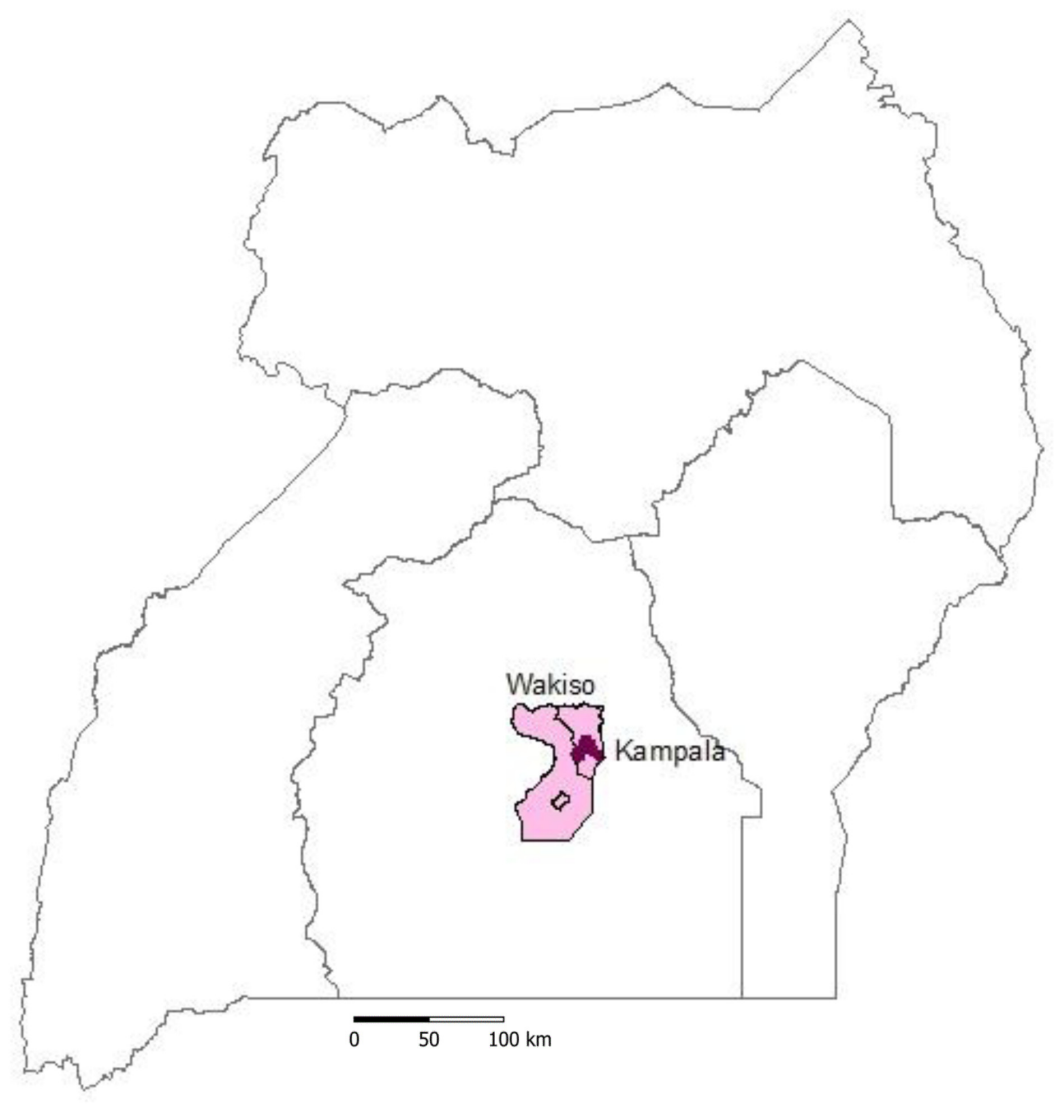
Map of Uganda showing the study area, Kampala the capital city of Uganda (dark colour) and Wakiso district (light colour).

**Figure 2.**
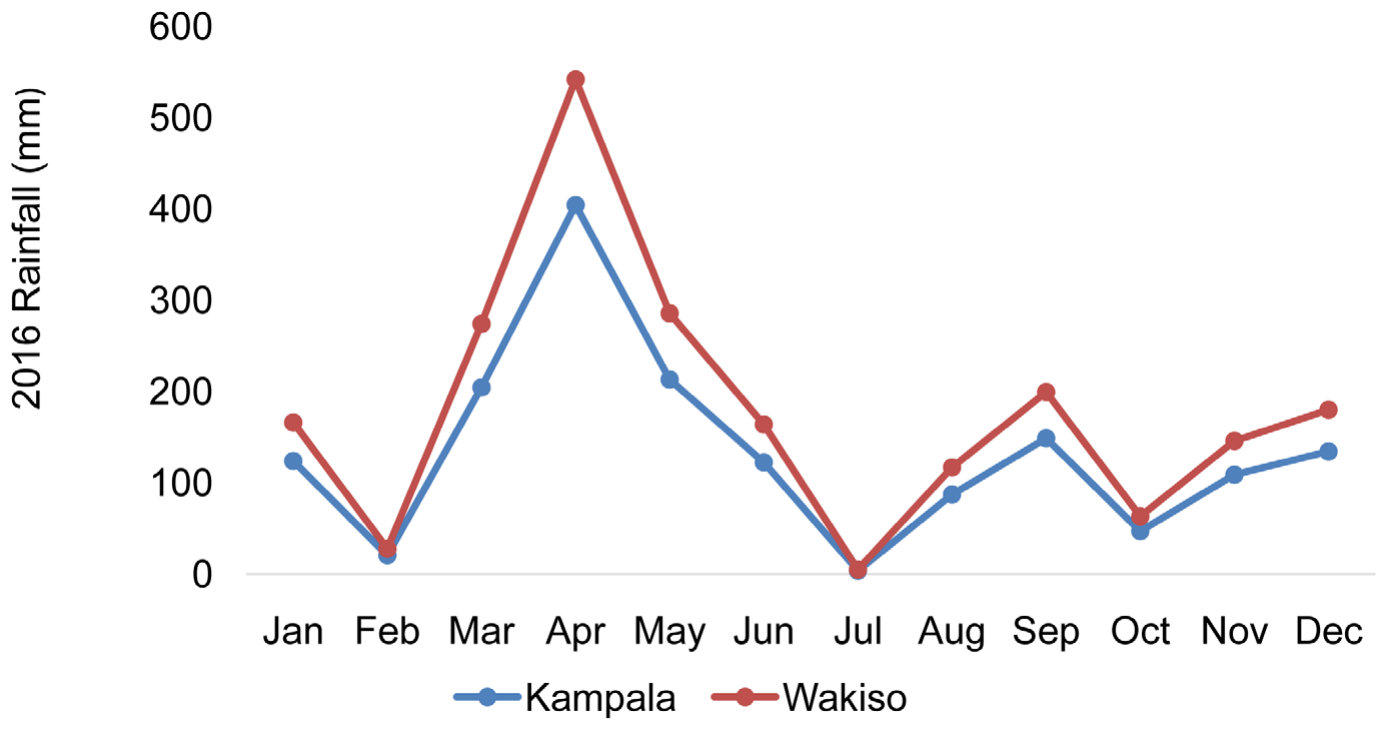
Rainfall pattern of Kampala and Wakiso district during the year 2016 ( UNMA, 2016).

 The study area was in Kampala City and Wakiso districts with a land area of 176 km ^2^ and 1906.7 km ^2^, respectively. Kampala (0°05′N–0°16′N and 32°30′E–32°38′E) is the capital city of Uganda. Wakiso (0° 24′ 0″ N and 32° 29′ 0″ E), at 59.2% urbanization level, is the largest urban district and surrounds Kampala in all directions ( Figure 1). The population of Kampala and Wakiso is approximately 1.5 million and 2 million individuals, respectively ( UBOS, 2016). Rainfall data for the year 2016 was obtained from Uganda National Meteorological Authority ( UNMA, 2016) ( Figure 2). 

### Landsat TM remote sensing data set

**Figure 3.**
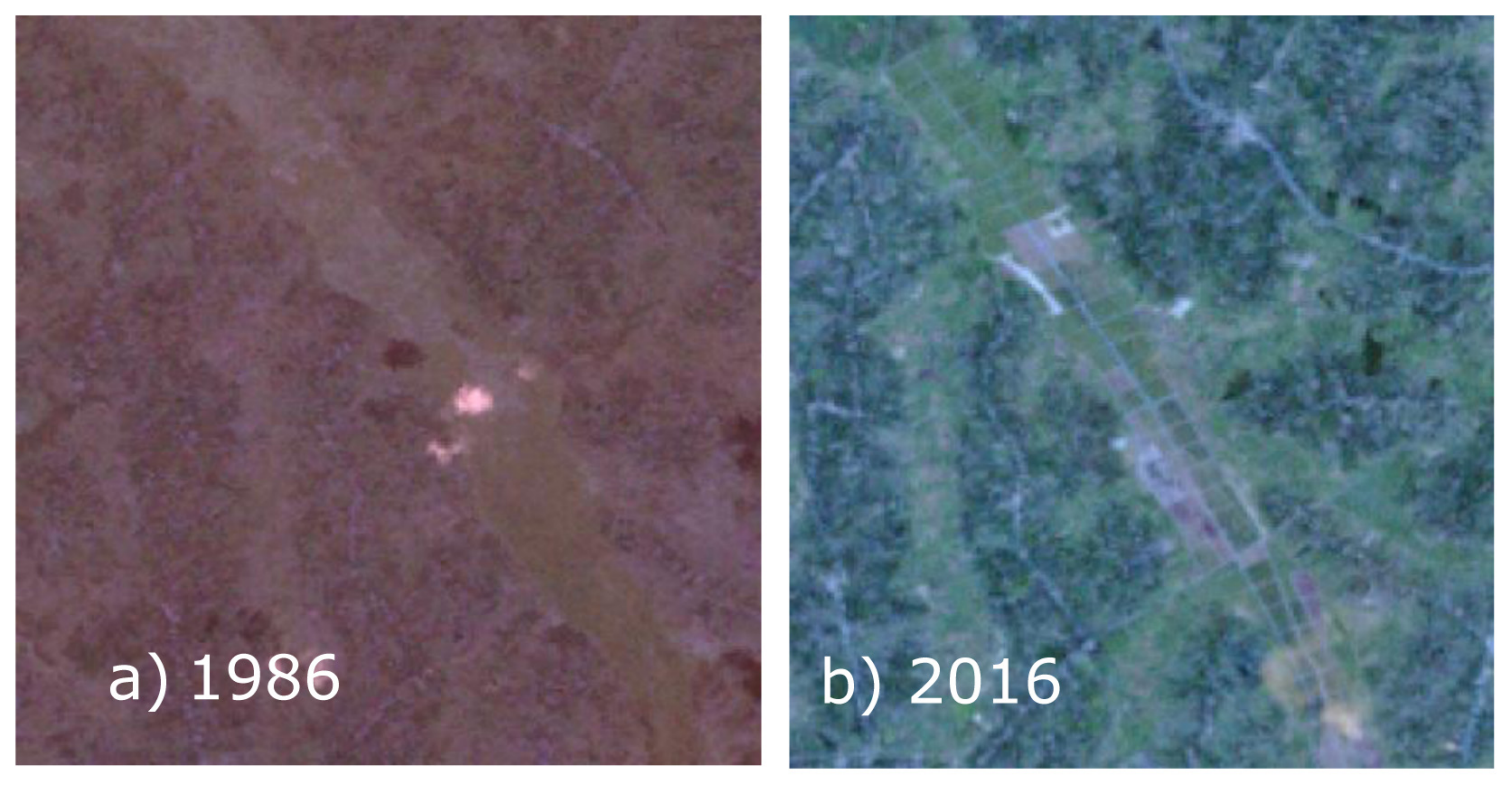
**a**) LANDSAT TM 1986 (Wakiso, area coverage 2000 pixels × 1800 pixels, Resolution 30m) and **b**) LANDSAT ETM 2016 (Wakiso, area coverage 2000 pixels × 1900 pixels, Resolution 30m) showing former wetlands ( **a**) converted to agriculture plots in ( **b**).

Remote sensing data were downloaded from https://earthexplorer.usgs.gov. Landsat image scenes (path 171, row 60, 30m resolution) were acquired for 1986 and 2016. The 1986 scene was from Landsat 5 Thematic mapper (TM), while that of 2016 was from Landsat Enhanced Thematic Mapper (ETM). The two images were geo-rectified with topographic maps and with 25 ground control points (GCPs). Ground Control Points are defined as points on the surface of the earth of known location used to geo-reference Landsat Level-1 data. These were identified from https://landsat.usgs.gov/gcp. ERDAS IMAGINE 9.3 software was used for geo-rectification. Alternative free software that can perform this task is BEAM, an open-source toolbox, and development platform for viewing, analyzing, and processing of remote sensing raster data ( https://earth.esa.int/web/sentinel/user-guides/software-tools/-/article/beam). Labels of classes used in this study included broad categories of land use and land cover, agriculture, forest, wetlands, and agriculture in wetlands ( Figure 3), built up and bare ground:

- Agriculture area: small plots of land or broad tracts of mechanized land areas;
- Forest classification: used training samples from Mabira forest (a natural forest), which was away from the study area on the satellite image, as there were no natural forests within the study area.
- Wetlands: marshland and seasonal ephemeral areas.

Since a previous data set for the 1986 scene was not available, classification was dependent on the cover types observed and the ground points (250) taken during fieldwork in 2016 (explained below). Agriculture, usually practiced on small plots of land with various crop mixtures with differing crop calendars, increased heterogeneity in agriculture pixels.

The limitation with the 1986 image was the large cloud cover specifically over the built-up Kampala area. With this observation, urban areas in the 1986 image were not classified. High-resolution images from Google, visual interpretation, ground survey, and familiarity of the study area were used to improve the accuracy of the classification. The accuracy of the classification results for 2016 Landsat TM images was assessed using 250 randomly sampled ground truth points, obtained from fieldwork (Explained below).

### MODIS surface reflectance 16-day composites

Free remote sensing data was obtained from the MODIS website: https://modis.gsfc.nasa.gov/data/dataprod/mod13.php. The data was visualized through the USGS Global visualization viewer (GloVis), an online search and order tool ( http://glovis.usgs.gov). GloVis was used to select satellite data for the area covering Kampala and Wakiso in Uganda. The MODIS Normalized Difference Vegetation Index (NDVI) collection 5 product MOD13Q1 was used in a hyper-temporal optical environment for stratification and crop characterization. MOD13 products estimate ground surface reflectance and are corrected for the effects of atmospheric gases and heavy aerosols. They are masked for water, clouds, and cloud shadows. MOD13 data was accessed as 16-day composites at 500-meter spatial resolution. Each pixel encompasses the best observation values within every 16 days. Each image includes a Red and Near-Infrared reflectance band. These bands are centered at 469, 645, and 858-nanometers, respectively, from which the NDVI ( equation 1) was calculated. MODIS 16-day composite images from April-September 2016 were used for deriving 15 NDVI images. The specific dates of the satellite: 2 ^nd^ and 18 ^th^ February, 5 ^th^ and 21 ^st^ March, 6 ^th ^and 22 ^nd^ April, 8 ^th^ and 24 ^th^ May, 9 ^th^ and 11 ^th^ June, 27 ^th^ July, 12 ^th^ and 28 ^th^ August, 13 ^th^ and 29 ^th^ September 2016. The choice of the dates was based on the availability of the satellite but followed the bimodal rainy season that starts from March to June and from August to November.

NDVI = ( *NIR* – *Red*)/( *NIR* + *Red*)             (1)

To generate the digital NDVI numbers (DN), linear stretching was applied. The minimum NDVI value, -1, was assigned 0 while the maximum NDVI value, 1, was assigned values from 1 to 255. The DN values were calculated as NDVI = 0.004 for DN – 0.1 ( FAO, 2017).

### MODIS-based stratification and characterization of urban agriculture in wetlands

***ISODATA Analysis.*** Using ERDAS IMAGINE 9.3 software, the 15 NDVI images were stacked one over the other in chronological order of the dates to create a hyper-temporal image data set (Alternative free software that can perform this task is BEAM, https://earth.esa.int/web/sentinel/user-guides/software-tools/-/article/beam). The hyper-temporal image data were then processed using an Iterative Self-Organizing Data Analysis Technique (ISODATA) and classified in 99 ISODATA runs outlining distributions of 2 to 100 classes. This was followed by a selection of the ideal number of classes using divergence separability statistics in a stratified random sampling ( Asilo *et al*., 2014; Girma *et al*., 2016). These were calculated such that the number of classes with the highest positive deviation from the trend line connecting classes was considered optimal ( Ali *et al*., 2013; Westinga *et al*., 2020). In this study, a total of 50 classes provided an optimal stratification for the NDVI-profiles.

***Cluster Analysis.*** Next, the class-specific NDVI-profiles were produced ( Khan *et al*., 2010; Nguyen *et al*., 2012) and plotted in Excel for visualization to give profile curves of NDVI Digital Number (DN) values for each 16-day composite from April to September 2016 (Provided as supplementary data, https://doi.pangaea.de/ 10.1594/PANGAEA.915587 [Kabiri *et al*., 2020e]). To obtain homogenous clusters from the NDVI-profiles, the 50 classes were processed using Hierarchical Cluster Analysis ( Ali *et al*., 2013; Westinga *et al*., 2020) using Pearson’s correlation as the proximity procedure. The clustering analysis was conducted in SPSS version 20.0 ( SPSS, 2011). A dendrogram of the clusters from the class groups was obtained at various hierarchical levels ( Figure 6). Hierarchical cluster analysis yielded a dendrogram ( Figure 6) that partitioned the 50 NDVI classes into 10 clusters ( Figure 7). 

***Fieldwork.*** To determine types of the crops growing in the wetlands and associated wetland encroachment associated with the 10 clusters segregated by the Hierarchical cluster analysis above, the 50 class-specific NDVI-profiles were vectorized. This yielded 50 vectors that were given a unique identifying colour and overlayed on a boundary map of Wakiso and Kampala. This vector data was input on ArcPad 10.3 on a Trimble GPS running ArcPad to be used for fieldwork to QGIS, Google maps app on an android phone can perform this task). In the period between April and December 2016, five sites (area) of each vector were visited giving a total of 250 sites (ground truth points) in Kampala and Wakiso. At each ground truth point, the following data was collected; XY coordinates, covering a percentage of vertical vegetation, dominant species, land cover, and land use. These gave an indication of the types of crops growing on the site and the associated wetland encroachment. Data collected from fieldwork was checked for completeness and was organized using the excel spreadsheet for analysis. Alternative software that can perform this task is QGIS, free and open-source software that can be downloaded at https://www.qgis.org/en/site/.

### Accuracy Assessment

Accuracy assessment of both the Landsat Imagery of 1986 and 2016 and the MODIS-derived urban agriculture was based on the kappa coefficient and confusion matrix assessing classified pixels with reference to 250 ground-truth points obtained from the fieldwork explained above. The ground observations obtained from fieldwork were used to characterize the similarities in the vegetation phenology represented by the 10 clusters and characterize crop agriculture in the wetlands.

## Results

### Rainfall

Usually, Kampala and Wakiso district located around the Lake Victoria basin is characterized by two rainy seasons. However, 2016 had one prominent rainy season that started in February, peaked in April (940 mm), and gradually dropped in July, which is a usual rain pattern in the first season of the study area. In the second half of the year, the rainfall pattern was more erratic with the amount of rainfall barely attaining 400 mm between September and December of 2016. This prominently dry season resulted in one of the worst droughts the country has faced in recent years. 

### The recent state of urban wetlands in Uganda

**Figure 4.**
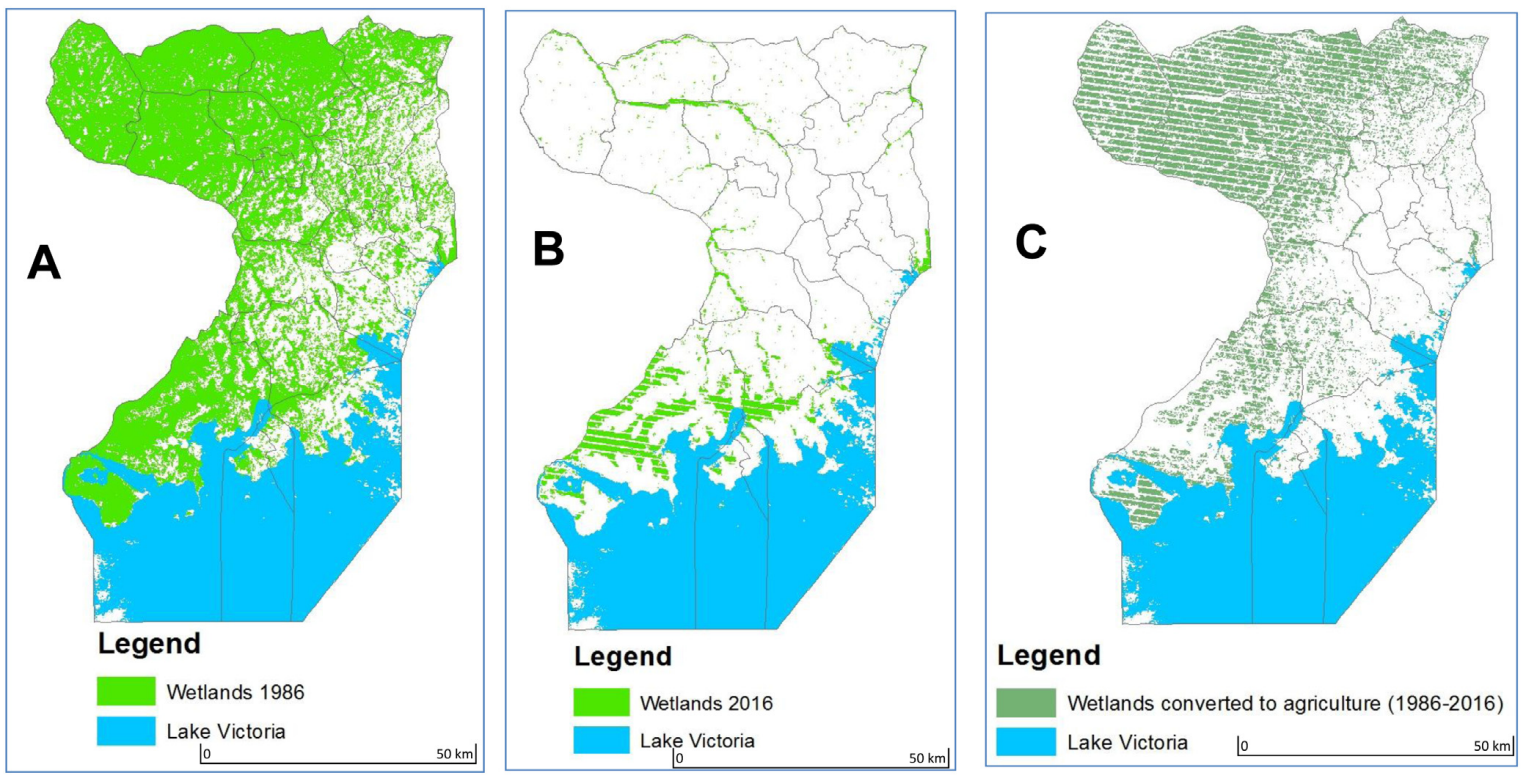
Landsat TM, 30-year land cover change showing wetland encroachment for agriculture in Wakiso and Kampala for agriculture from 1986 ( **A**), 2016 ( **B**) and wetlands converted to agriculture between 1986 and 2016 ( **C**).

The overall classification accuracy and Kappa coefficient for 1986 and 2016 land cover maps from the Landsat TM remote sensing data of the Wakiso-Kampala study area was, 83.1% and 0.87, and 87% and 0.85, respectively. We found that over 30 years, 72,828 ha of the Wakiso-Kampala wetlands, have been lost ( Figure 4 and Figure 5). Agriculture on the other hand doubled in cultivation area. Of the new cultivation area, 16,488 ha have been reclaimed from wetlands. The overall accuracy and Kappa coefficient for the MODIS-based stratification Wakiso-Kampala study area for crop agriculture in the wetlands was 92% and 0.93, respectively. Our results showed that all crop agriculture segregated in Kampala using hyper-temporal remote sensing was in the wetlands, while 73% of the crop agriculture segregated in Wakiso was in the wetlands. 

**Figure 5 (a).**
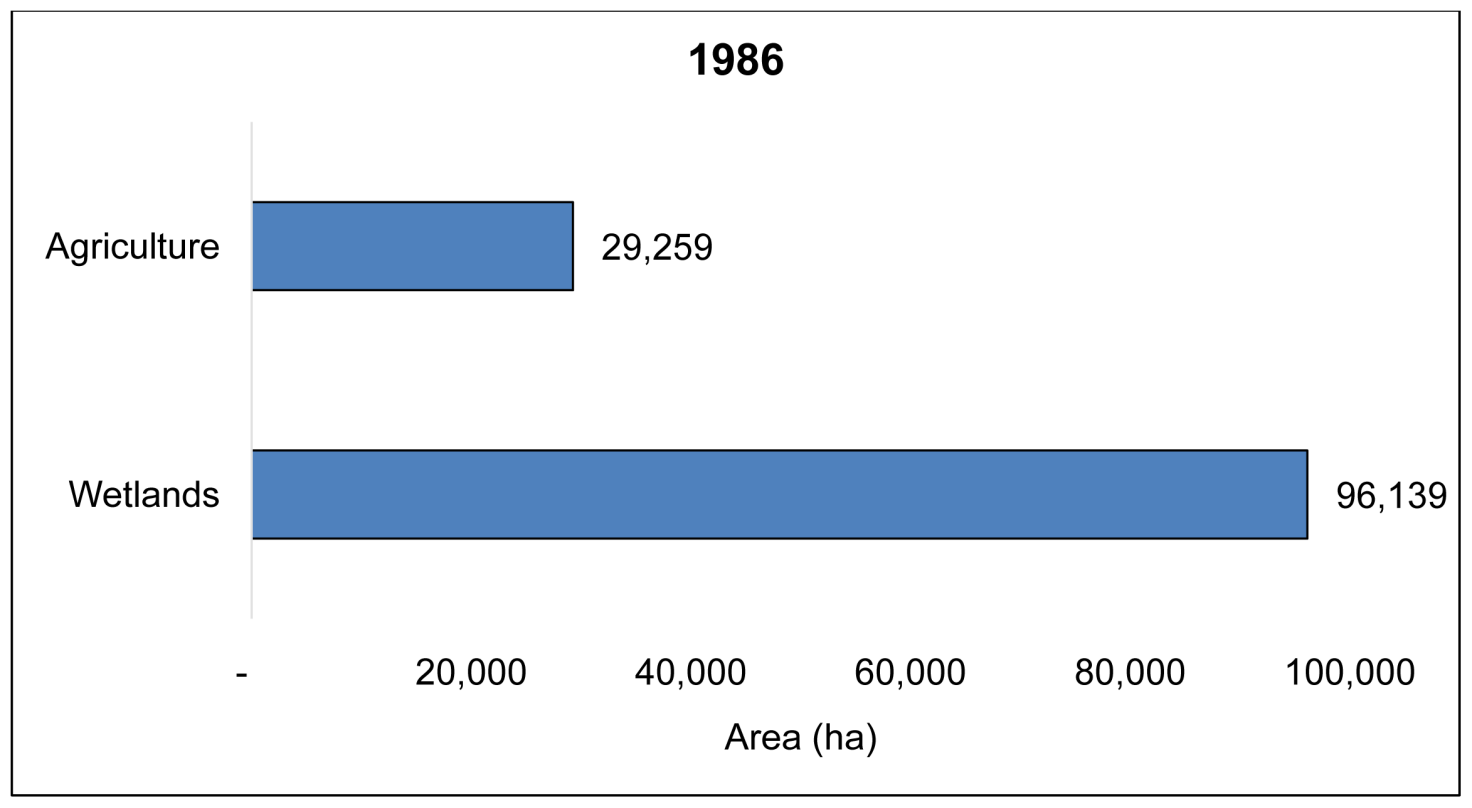
Wetlands and agricultural land cover in Wakiso-Kampala study area in 1986.

**Figure 5 (b).**
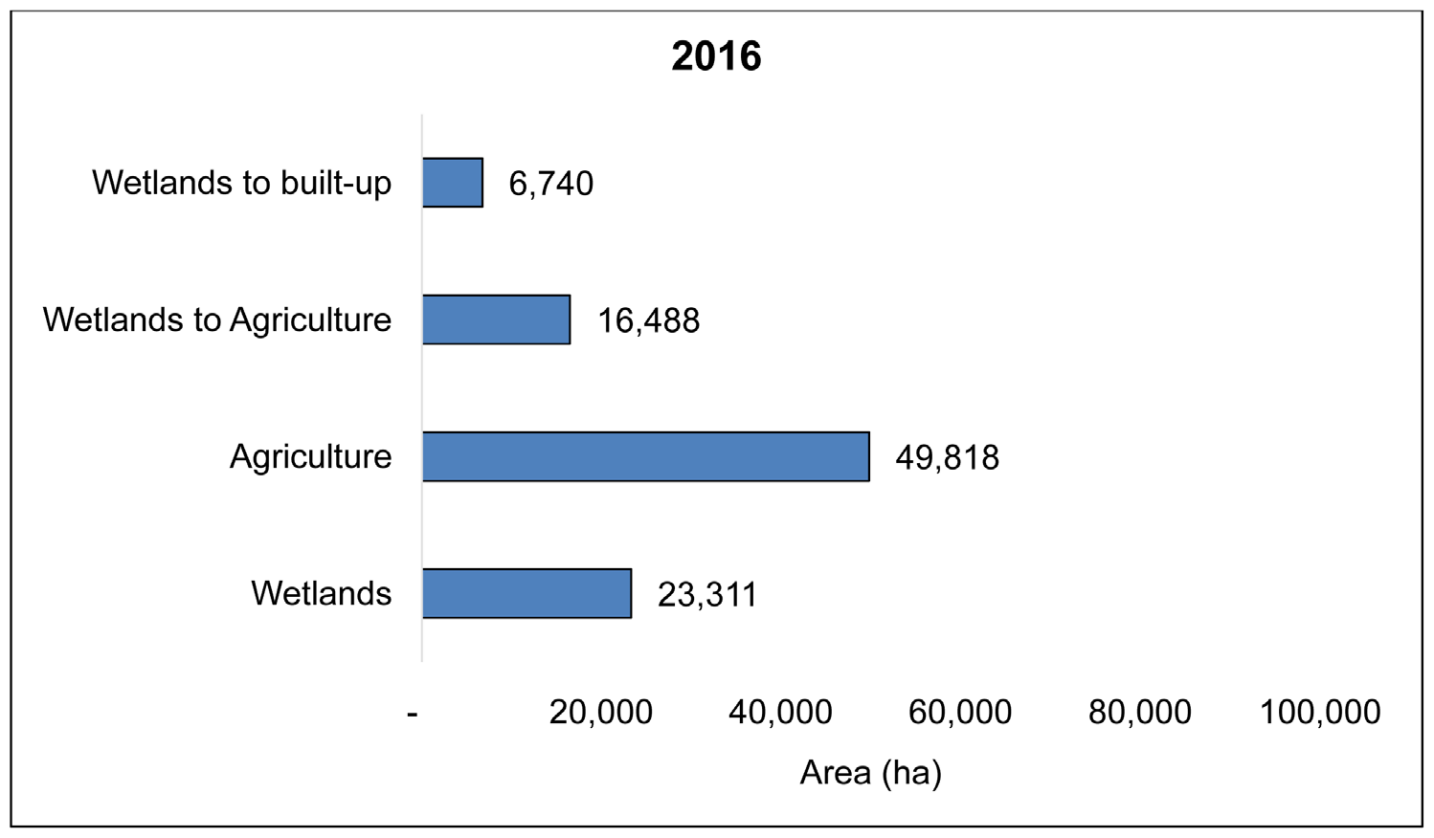
State of wetlands and agriculture in 2016 and the level of wetland encroachment for agriculture and built-up in Wakiso and Kampala study area.

**Figure 6.**
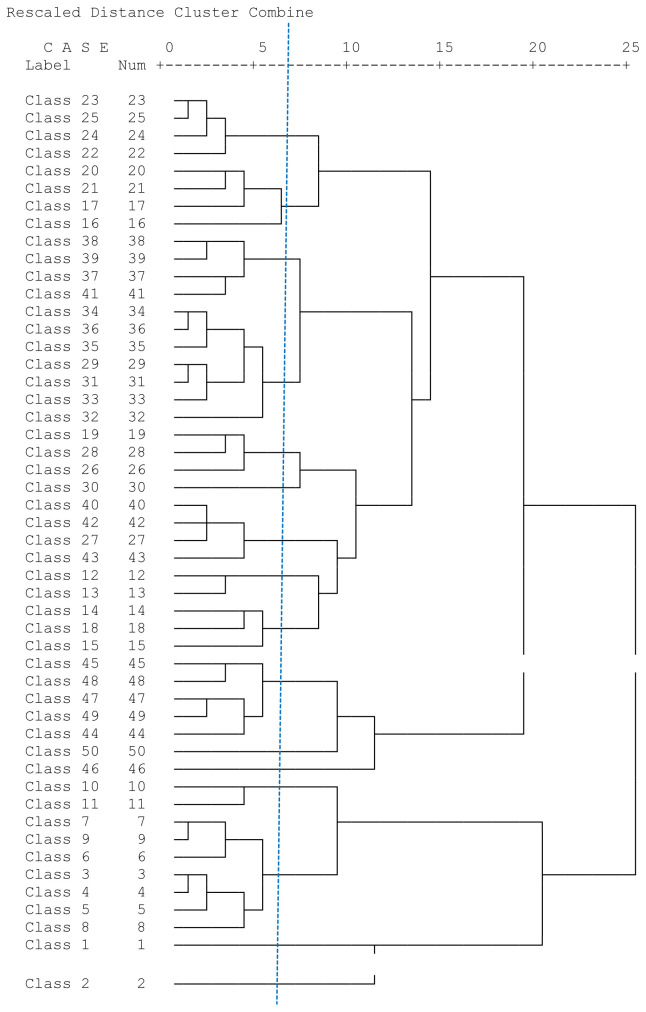
Dendrogram showing the grouping of 50 NDVI classes segregated by Hierarchical cluster analysis.

**Figure 7.**
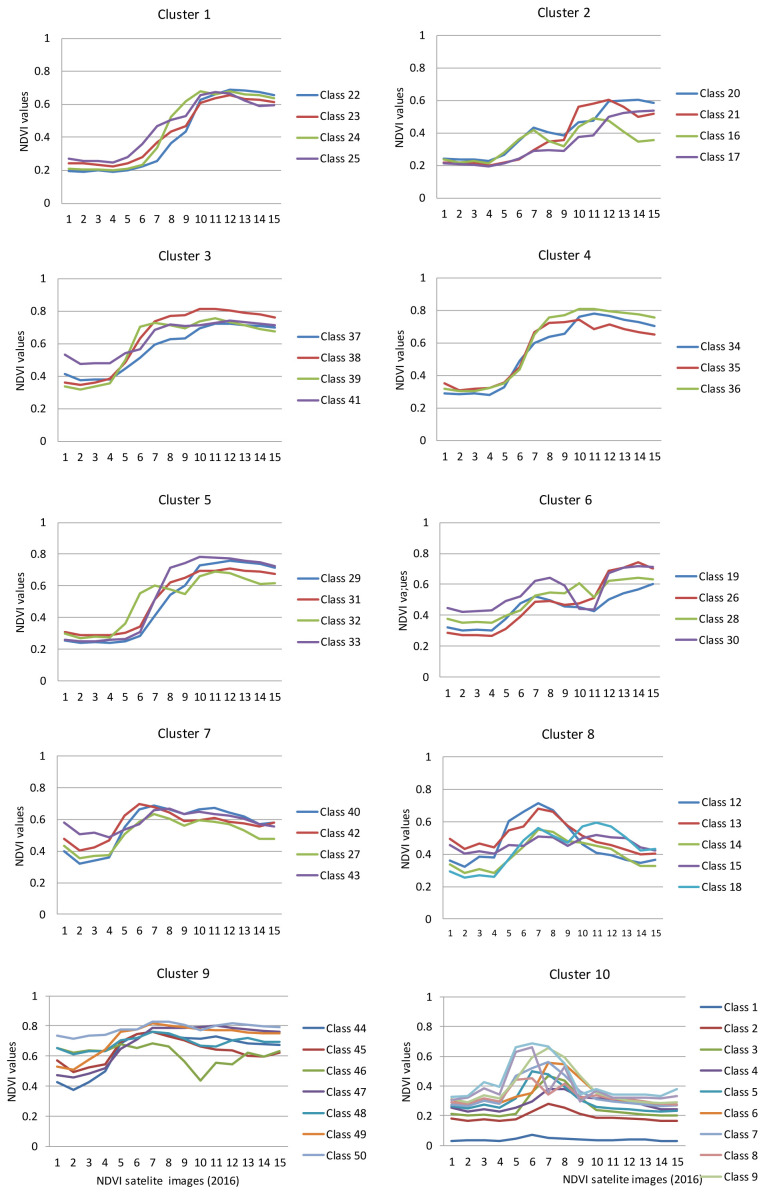
Respective profile curves of NDVI plotted from clusters produced by the dendrogram in Figure 6. Numbers 1 to 15 in the legend refer to the chronology of dates of the satellites taken every 16 days in the two 2016 growing seasons of Kampala and Wakiso.

### Characterization of crop agriculture in wetlands

Five of the clusters (clusters 1–5) showed sigmoid curves of NDVI values as the year progressed. These five clusters appeared similar but differed in values at the beginning of the year and the levelling off points. The NDVI profiles in cluster 1 (classes 22–25), started at 0.2- 0.3 values and levelled off between 0.7–0.6 values, while the profiles in cluster 2 (classes 16, 17, 21 & 20), also started at 0.2–0.3 values but levelled off between 0.6–0.5 values but were more erratic in shape. The NDVI profiles in cluster 3 (classes 37–39 & 41), started at 0.35–0.55 values, levelled off below 0.8 while the NDVI profiles in cluster 4 (classes 34–36) started at 0.5 values, also levelled off below 0.8 values but had a smoother sigmoid growth than cluster 3. Cluster 5 (classes 29, 31–33) was similar to cluster 3 but differed by starting at 0.3 values and had steeper growth than cluster 3. Clusters 6, 7, and 8 were similar but differed in their exhibition of prominent peaks during the year. The NDVI profiles in cluster 6 (classes 19, 26, 28 & 30) started at 0.3 values but peaked in early May just above 0.6 values, dipped deeply in early July (just above 4 values), and peaked again in September (about 7 values). The NDVI profiles in cluster 7 (classes 27, 40, 42 & 43) started between 0.4–0.6 values but peaked in early May just above 0.6 values, slightly dipped in early June (just below 0.6 values), and peaked again in late July (about 0.65 values). The NDVI profiles in cluster 8 (classes 12–15, & 18), on the other hand, started between 0.25–0.5 values, prominently peaked in early May (just above 0.6 values), strongly dropped in early July (just below 0.6 values) and conspicuously peaked again in mid-August but at lower values (below 0.6) than they did in May. The NDVI profiles of cluster 9 (classes 44–50) were flatter throughout the year except for the NDVI profile of class 46 that prominently dipped in early July but rose gradually during the last quarter of the year. The NDVI profiles of cluster 10 (classes 1–10) were conspicuously different from all the other nine clusters in that the first quarter of the year started with flat profiles ranging between 0.18-0.38 values (except class 1). However, in late March, the profiles rose drastically to just above 0.6 values and peaked in late April and then gradually dropped to early July and then remained flattened out for the rest of the year.

**Figure 8.**
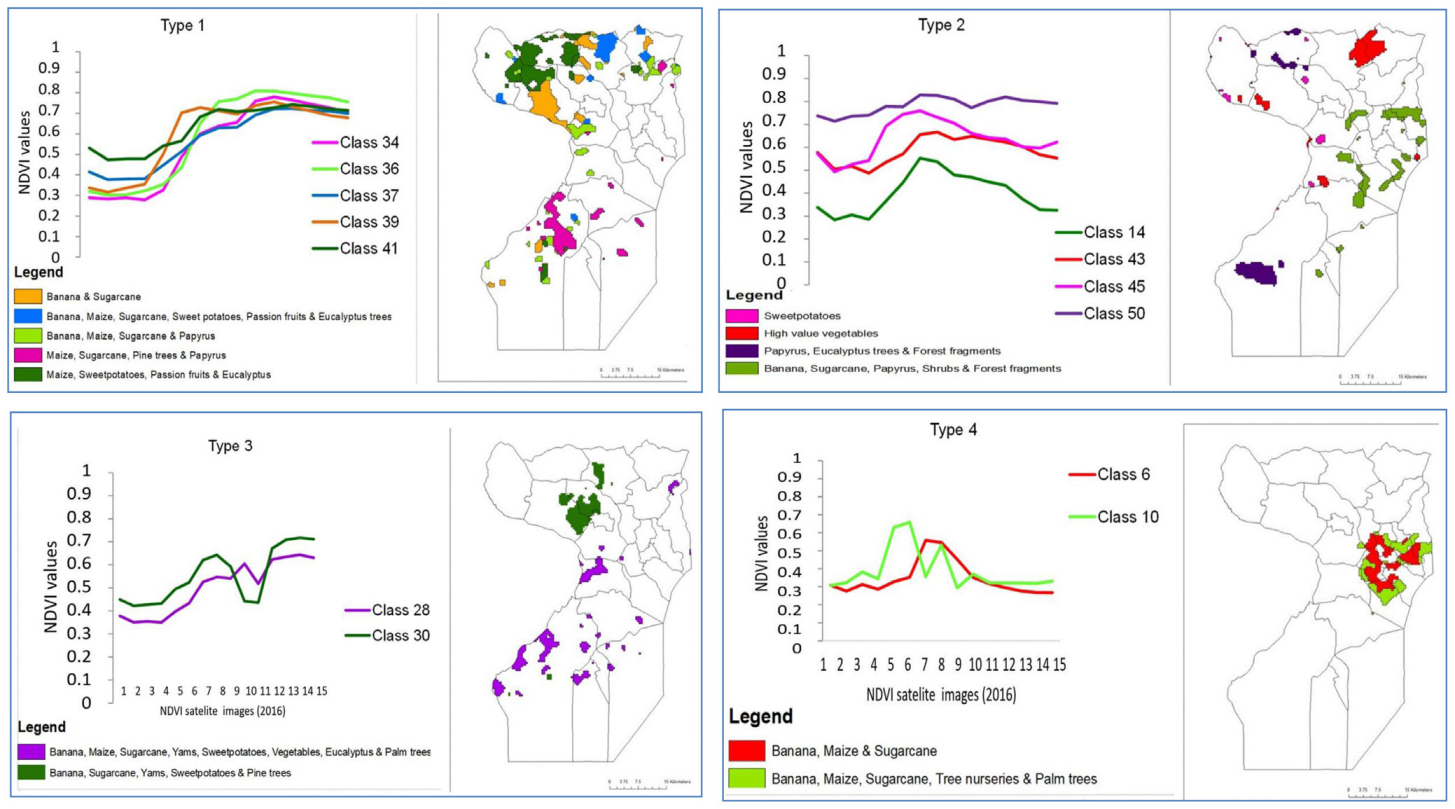
Types of crops and crop mixtures in Uganda’s urban wetlands. On the right is a Wakiso-Kampala map showing the land area corresponding to respective crops and crop mixtures in a similar colour.

**Figure 9.**
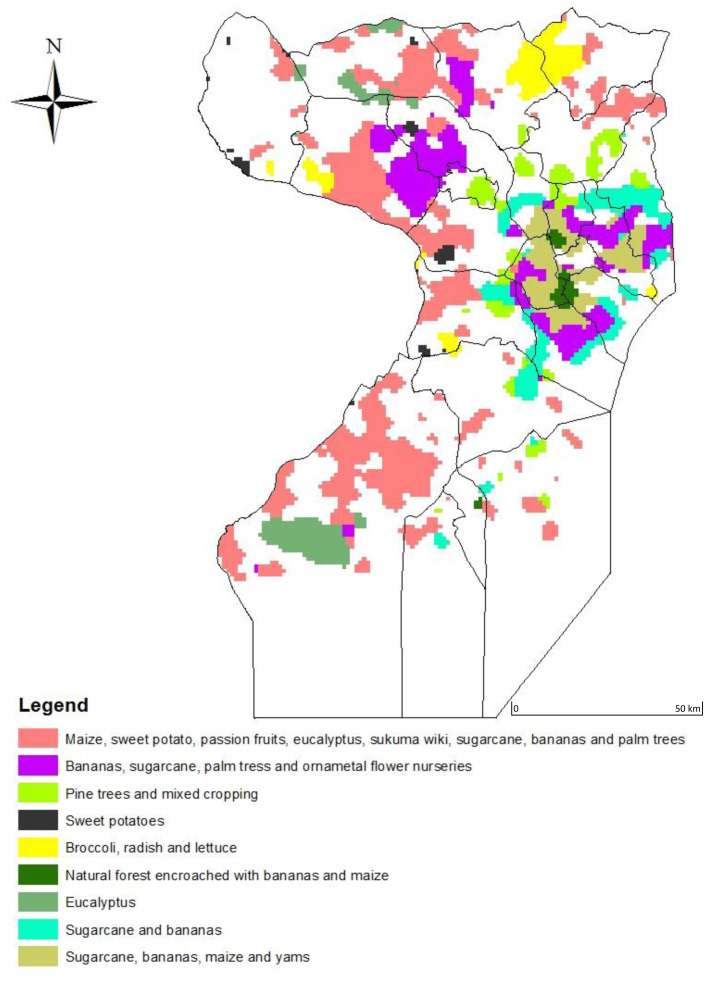
A map showing the land area extent of wetland encroachment for agriculture and agroforestry in urban and per-urban Kampala and Wakiso in Uganda.

Ground observations found that of 50 classes represented by the 10 clusters, 13 of them belonged to agriculture in the wetlands. The wetland classes included classes 6, 10, 14, 28, 34, 36, 37, 39, 41, 30, 43, 45 and 50. These were represented in all clusters except clusters 1, 2, and 5. The major crops grown in these urban wetlands in order of frequency were banana (20%), sugarcane (22%), maize (17%), *Eucalyptus* (12%), and sweet potatoes (10%), while ornamental nurseries, pine trees, vegetables, and passion fruits were each at 5%. Using visual interpretation, the 13 classes in the wetlands were graphed using similarity of the shape NDVI profiles, which yielded 4 categories of plant/crop types. Type 1 included NDVI classes 34, 36, 37, 39 and 41, while Type 2 included 14, 43, 45, and 50. Type 3 included classes 28 and 30 while Type 4 included classes 6 and 10. The crops and crop mixtures that each of these classes represent are shown in maps in Figure 8. The land area extent of wetland encroachment for agriculture and agroforestry in urban and per-urban Kampala and Wakiso in Uganda in Figure 9.

## Discussion

Our results reveal that 76% of wetlands in the Wakiso-Kampala study area have been lost. Of the lost wetlands, 23% have been converted to agricultural cultivation area. The MODIS Collection 5 land cover datasets of 500 m resolution processed with the ISODATA clustering algorithm served the purpose of using remote sensing data for monitoring wetland encroachment by crop agriculture. The 13 classes of NDVI profiles identified in the wetlands seemed to be determined by the type of agriculture practiced in these wet ecosystems. The vegetation in Type 1 was prominent in western Wakiso in the sub-counties of Kakiri, Masuliita, Gombe, and Kasanje whose wetlands are made of papyrus flora, which is the natural wetland vegetation. The NDVI profiles in this type had sigmoid curves that rose in early April at the peak of the rainy season, depicting a crop phenology that followed the rains or one that responded strongly to high moisture. This assertion was confirmed by ground data that observed that plant/crop Type 1, was dominated by perennial crops (bananas: *Musa* spp; sugarcane: *Saccharum officinarum*) *,* seasonal crops (specifically maize: *Zea mays*), fruit farming (specifically passion fruit: *Passiflora edulis*) and silviculture (pine trees: *Pinus* spp. and *Eucalyptus* spp.). The vegetation in Type 2 was prominent in Wakiso in sub-counties of Busukuma, Kiira, Sbagabo-Makindye, and Kasanje which are more peri-urban sub counties with a large influence of Kampala city in terms of urban markets. The profiles in Type 2 were flatter curves depicting a continuous crop phenology that can either represent uninterrupted crop growth or perennial crops. Field observations found that Type 2 was dominated by high-value vegetables (radish: *Raphanus* spp.; broccoli: *Brassica oleracea;* lettuce: *Lactuca sativa*; sweet potatoes: *Ipomoea batatas)*, which are constantly cropped, and perennial crops such as banana and sugarcane. This finding is indicative of the advantage of the constant moisture supply provided by these wetlands. The vegetation in Type 3, was prominent in Wakiso in the sub counties of Kasanje, Wakiso Town council, Kakiri, and Gombe. The profiles in Type 3 rose sharply in early April, depicting vegetation response to rainfall and fell in early July portraying harvest, but rose sharply again in late July, representing the commencement of a second cropping season. Field observations found that Type 3 was a mixture of crops found in both Type 1 and Type 2 that were perennial, annual, silviculture, and horticulture, still indicating the extent to which urban wetland ecosystems offer a substantial supply of moisture to carry two cropping seasons. Type 4 was prominently in the wetlands of the Greater Kampala metropolitan division of Nakawa, Rubaga, Makindye, and the Central city area. The most prominent crops were banana, maize, sugarcane, and tree nurseries.

We also observed that some NDVI profiles in wetland classes levelled off during July and plateaued through the rest of the year. This implied that the crops grown in the wetlands responded strongly to the rainfall season but also remained thriving during the prolonged drought. Although most of the country suffered an acute food insecurity situation, which saw Uganda lose her food secure status ( OPM, 2017), crops in these urban wetlands exhibited resilience, owing to the moisture retained in these wetter ecosystems; wetlands are linked to the accumulation of fertile sediment during floods and long periods of water retention ( Dixon & Wood, 2003). Our ground-based observations from collecting training datasets of individual crops grown in the wetlands during fieldwork were key to ascertaining the results of ISODATA clustering of NDVI profiles. Seasonal variations such as erratic rainfall patterns observed in the study period can add to the difficulty of detecting phenological changes ( White *et al*., 1997). Currently, there is limited biome-scale ground phenological data of Uganda’s urban wetlands from previous years. The lack of biome-scale ground can inhibit the effective assessment of satellite data where remote sensors integrate pixel areas larger than 250m ^2^ ( Reed *et al*., 2003). Advances in remote sensing have found a way out of this challenge by quantifying urban-rural phenological differences with temperature components and genetic variance of species communities ( Kathuroju *et al*., 2007; Zhang *et al*., 2004). Recently, a method of object-based crop identification using multiple vegetation indices, textural features, and crop phenology was demonstrated to successfully interpret crop identification ( Peña-Barragán *et al*., 2011).

The technique we have shown in this study is a reproducible map-making method that can enable the national environment system of Uganda across sectors, to improve the ability to quickly identify wetland encroachment. It can serve as an early warning system that can minimize the loss of the remaining wetlands. The moderate resolution (500m) remote sensing repeated at frequent fortnight intervals could enhance such a monitoring system that would respond in near real-time. Our study offers a baseline for the leftover wetlands by 2016, against which identified changes can be suitably interpreted. In the face of climate change, it will be essential to combine these remotely sensed data with temperature, precipitation, soils, and topographic information ( Hargrove *et al*., 2009) to interpret the changing environment of the wetlands on the ground. However, this implies handling of enormous data volumes requiring enhanced capacity building in large data computing urban planning.

 We recommend that the dynamicity of Uganda’s urban wetland implies that successful implementation of wetland monitoring will require the deployment of an operational observation and monitoring system strategized on three scales, 1) satellite-based monitoring of urban areas to detect specific locations where encroachment is suspected, 2) a more refined resolution consisting of airborne drones and on-ground monitoring to evaluate the warning from the satellite-based monitoring to detect wetland encroachment, and 3) Citizen policing where the population detects any wetland encroachment through real-time camera recording and share on social media using mobile phones. These three scales of wetland monitoring if consistently utilized, have the potent cost-effective effective and efficient. In addition, this will create a record of long-term monitoring that will become an invaluable vital reference longer-term variations can be detected. Our urban wetland phenology data set is available for distribution and we encourage its use and exploration of its utility with us.

### Policy implications

Our results clearly show that wetlands in Uganda’s urban areas have been the prime target for agricultural expansion in the last three decades. The maps show that Uganda’s urban wetlands have been disappearing at a rate of 2,500 ha per year implying that the ecosystem services provided by these wetlands have been lost. At this rate, there will be no more wetlands left in Wakiso and Kampala by 2029.

The dynamic situation of these urban wetlands requires an informed understanding of the ecological and socio-economic benefits that they provide. There is a need to recognize the longer-term degradation threats and more spatially extensive impacts of these changes. This calls for coordinated adaptation strategies between scientists, policymakers, and urban dwellers for equitable utilization of wetlands without compromising their ecosystem services and economic benefits. Some studies have shown that urban wetlands in Uganda contribute approximately US $432 per year to local communities practicing subsistence agriculture ( Turyahabwe *et al*., 2013). Moreover, the type of crops grown in the urban wetlands are important Ugandan staple crops that have a high economic value in urban markets but are also a reflection of urban nutritional combinations. It is not surprising that bananas dominated urban wetland agriculture, as these are the country’s staple crop. Sugarcane and fresh roasted maize are enjoyed by urban dwellers as snacks and are sold along roadsides. In other countries in sub-Saharan Africa, an increasing population in combination with efforts to increase food security has intensified pressure to expand agriculture in wetlands. For instance, in many parts of eastern and central Africa, it has been observed that up to three crops per year can be grown in wetlands significantly contributing to food security. For example, in Tanzania, the Kilombero wetland was found to contribute up to 98% of food intake for all households surveyed irrespective of socioeconomic status ( Rebelo *et al*., 2010).

It has been suggested that wetlands can be converted to include intensification of a specific wetland strategy, such as the complete reclamation or commercial agriculture or industrial development, which are considered to be more economically viable ( Hollis, 1990). Conversely, it has been a matter of debate whether quantifying the economic value of wetlands in Africa undervalues their importance for their future utilization ( Schuyt, 2005a; Seyam *et al*., 2001). For example, already, the essential role that wetlands play in regulating the flow of water into Lake Victoria has been lost ( Olindo, 1992). A couple of decades ago, agriculture was responsible for 80% and 75% of riverine phosphorus and nitrogen entering Lake Victoria ( Odada *et al*., 2004; Scheren *et al*., 2000). Papyrus wetlands play a significant role of filtration and protection of the lake from eutrophication acting as sediment traps and buffer discharges ( Ryken *et al.*, 2015). Whereas short-term impacts are already visible, studies on the long-term impacts of such massive wetland encroachment at both local and regional scales are limited. The danger is that the current wetland exploitation for food security may be a trade-off between the provision of food in the short term and the loss of important ecosystems services in the long term. This points to the urgent need by the Government of Uganda to increase funding for wetland reclamation programs to restore and reconstruct lost and fragmented wetlands.

Map developed our study clearly shows that a large demographic of urban dwellers are using wetlands for food security and poverty eradication. This implies that there lies a dilemma in implementing wetland conservation acts in the same framework as the poverty eradication policies. Poverty eradication policies conflict with wetland conservation policies. It is therefore not surprising that despite the existence of seven policies protecting wetlands, enforcement and compliance systems have not been suited for the dynamicity of urban growth. This calls for ministries responsible for the operation of these two policies to harmonize implementation to find a middle ground to manage, restore, reconstruct or reclaim these urban wetlands. One way to harmonize these conflicting policies is to develop strategies that are inclusive of challenges faced by the urban poor while at the same time minimizing the pressures on urban environments. For instance, we could not ascertain the fraction of commercial from subsistence farming that was provided by these urban wetlands as it was not easy to identify owners of the crops in the wetlands. It is possible that urban dwellers farming in wetlands are aware that it could be illegal, but do not understand the framework of the impropriety; they take care of crops very early in the morning and then have other occupations during the day. Policy regulators on the other hand observe growing crops but cannot identify the owners or whether the agricultural practice used is suitable for wetlands. This indicates that there lacks sensitization of simple but precise indicators of what wetland encroachment for agriculture is to laypersons. In addition, government protection dialogue with relevant stakeholders is rather high-handed (e.g. destroying food crops grown in wetlands or forceful evictions) ( DISPATCH, 2019; Monitor, 2017).

The tendency to emphasize discipline-bound legislations could easily have demoralized citizens from recognizing potential economic and ecosystem services of these urban wetlands. This has in turn undermined the conservation of biodiversity and weakened protection laws. At the same time, wetlands are seen as an easy option for the construction of infrastructure. For instance, in recent years, to avoid compensation to evacuated urban settlements on road reserves, major roads have been constructed in the middle of papyrus wetlands. In return, flood events have increased in adjacent areas that are hazardous to the most vulnerable urban poor ( ADB, 2008; Isunju *et al*., 2016b). Already, in the Wakiso district, wetland encroachment for settlement and agriculture has changed the local area climate in terms of increasing drought, reductions in rainfall seasons, and increasing day and night temperatures ( WDLG, 2017). This may have far-reaching consequences to local communities dependent on these wetlands but has significantly contributed to the environmental crisis in the Lake Victoria basin. For instance, it has been found that a wetland must maintain a connection with a Great Lake to promote and enhance efficient fish utilization of the high productivity of wetland vegetation and that additional resilience is provided to fish species that spawn in wetlands since they can produce two cohorts (one in wetlands and one in the Great Lakes), and that fluctuating water levels are important in sustaining habitat diversity and productivity ( Jude & Pappas, 1992). The Kampala-Wakiso wetlands are connected to Lake Victoria for this very ecosystem service. However, the level of degradation and encroachment has led to wetland fragmentation. It is not surprising therefore that in recent years, Fish species in Lake Victoria have declined. In addition to overfishing, the destruction of these fish breeding areas could have contributed to this decline ( Njiru *et al*., 2010).

The significant challenge in the implementation of policies that effectively protect these wetlands is that legislative and policy provisions have lagged behind growing scientific knowledge and understanding. Matching policy to cutting-edge science can minimize and mitigate the impacts on ecosystems resulting from overexploitation ( MacKay, 2006). Government environmental protection bodies have access to widely applied and tested methods of assessing wetland encroachment at larger scales, such as remote sensing data ( WETLANDS-ATLAS, 2016). These institutions can integrate regional and local databases to identify potentially vulnerable wetland-dependent ecosystems. Scientists on the other hand can develop scientific knowledge on understanding wetland-dependent ecosystems at both local and regional scales. When these two levels of understanding are merged, this information can be useful in the implementation and strengthening of already existent but poor policies. For example, modelling scenarios of threatened and vulnerable ecosystems to policies can be used to predict the future of wetland encroachment as evidence-based data to strengthen weak policies ( Odgaarda *et al*., 2017). In addition, the ability to address multiple approaches that identify the various ecosystem services provided by wetland ecosystems through rapid assessment of wetland ecosystem services is required. These can provide an output of a range of ecosystem services through a rapid and comprehensive overview of the various benefits provided by wetlands ( Everard & McInnes, 2013; McInnes & Everard, 2017). The Rapid Assessment of Wetland Ecosystem Services (RAWES) approach interactively involves all stakeholders and equips wetland managers to address data constraints about the magnitude and extent of beneficiaries. The benefits are linked through three scales: local benefits (at household and individual level), regional benefits (at wider catchment levels), and global benefits (those beyond national boundaries) ( McInnes & Everard, 2017). Such an approach could sufficiently increase the ability to recognize the importance of ecosystem services, monetary valuation, and multiplicity of social-economic benefits of these urban wetlands. 

The level of wetland degradation revealed by this study shows that protection of urban wetlands has been relatively low pointing to poor policy implementation over the years. This study has demonstrated that despite environmental data being scarce and heterogeneous landscapes in Africa being difficult to map, wetland regulators in Uganda can utilize free remote sensing data to monitor wetlands. In addition, hyper-temporal remote sensing of urban wetlands can be a cost-effective method of monitoring wetland encroachment through the provision of consistent temporal records.

## Conclusion

The average rate of loss of the Kampala-Wakiso wetlands over the past 30 years has been nearly 2500 ha annually, although the actual rate of loss has likely been variable from year to year according to economic and policy influences. It is possible, however, that by 2029 no wetlands will remain in the Kampala-Wakiso area. The technique we have shown in this study is a reproducible map-making method that can enable the national environment system of Uganda across sectors, to improve the ability to quickly identify wetland encroachment for urban agriculture. It can serve as an early warning system that can minimize the loss of the remaining wetlands. The moderate resolution (500m) remote sensing repeated at frequent fortnight intervals could enhance such a monitoring system that would respond in near real-time. Our study offers a baseline for the remaining wetlands by 2016, against which identified changes can be suitably interpreted. Policies should shift to include a long-term sustainability focus that allows conservation of the Kampala-Wakiso wetlands without which ecosystem services will decline and ultimately impact water quality improvement, flood abatement, carbon sequestration, biodiversity ecological units of wildlife, and medicinal plants. In addition, policymakers should merge conflicting policies between ministries promoting food security and poverty eradication with ministries regulating wetlands.

## Data availability

### Underlying data

Remote sensing data: https://earthexplorer.usgs.gov

MODIS remote sensing data: https://modis.gsfc.nasa.gov/data/dataprod/mod13.php

This project contains the following underlying data:

1. Satellite imagery from LAND SAT (1986 and 2016).
2. Satellite imagery from SPOT NDVI (monthly 2015-2016).
3. ISODATA clustering of NDVI profiles for 50 classes and wetland class shapefiles for wetland encroachment for the last 30 years.
4. Ground truth points for Kampala and Wakiso wetlands
