# Supplementary material for: Detecting wetland encroachment and urban agriculture land classification in Uganda using hyper-temporal remote sensing
Source: AAS Open Res. 2022 Feb 16;3:18. Originally published 2020 May 12. [Version 2] doi: 10.12688/aasopenres.13040.2 (PMC10521074; doi:10.12688/aasopenres.13040.2)
Supplement: Peer review history

# Reviewer report: Reviewer response for version 2

**DOI:** 10.21956/aasopenres.14490.r30127

**Published:** 2023-09-07

**Authors:** Heng Wan

**Affiliations:** 1 Pacific Northwest National Laboratory, Richland, USA

The manuscript entitled “Detecting wetland encroachment and urban agriculture land classification in Uganda using hyper-temporal remote sensing” quantified urban wetland losses in Uganda based on Landsat images, MODIS 16-day composites product, and ground observations. The manuscript is well-written; however, some issues need to be addressed before indexing:

1. The author implemented image classification for the two Landsat images, however, no detailed classification algorithm is illustrated in the method part.
2. ISODATA analysis is generally applied to one image (obtained from a single time point) to group similar pixels together based on their spectral similarities. The authors seem to have applied ISODATA analysis on the NDVI time-series data, and I am wondering how the algorithm works here.
3. In figure 7 cluster 10, the blue curve having a stable NDVI value close to 0. Is this related to non-vegetation surface? I am wondering whether the NDVI-based clustering is done for all the pixels in the study area, or just done for wetland pixels obtained from the previous step (Landsat imagery-based classification).
4. “To determine types of the crops growing in the wetlands and associated wetland encroachment associated with the 10 clusters segregated by the Hierarchical cluster analysis above, the 50 class-specific NDVI-profiles were vectorized.” What does “vectorize” here mean? Do the authors mean that for each class, the corresponding pixel clusters are vectorized? If so, are the authors specifically targeting any large clusters exceeding a certain threshold (e.g., pixel cluster for class 1 exceeding 100 pixels)?
5. The author identified 13 agricultures in the wetlands in the 10 clusters. What are the remaining 37 classes?
6. The “cluster analysis” seems redundant in this study. If we remove this analysis and do not cluster the 50 classes into 10 clusters, we could still conduct field work for the corresponding 50 vectors, and then identify the agriculture types in the wetland and continue the following analysis. Can the authors justify the implementation of cluster analysis here?

There are some minor issues:

1. “To generate the digital NDVI numbers (DN), linear stretching was applied”. What is digital NDVI number (DN)? Are you referring to the calculation of NDVI values based on digital numbers of the MODIS red and near infrared band? Also, I don’t quite understand this sentence: “The DN values were calculated as NDVI = 0.004 for DN – 0.1”.
2. “The NDVI profiles in cluster 6 (classes 19, 26, 28 & 30) started at 0.3 values but peaked in early May just above 0.6 values, dipped deeply in early July (just above 4 values), and peaked again in September (about 7 values)”. The values of 4 and 7 in the parenthesis should be 0.4 and 0.7. Also, you may delete the “values” after each number.
3. Based on my understanding, figure 9 shows all the wetland encroachment for agriculture and agroforestry by different vegetation mixture categories, which should be the aggregated results from all the subplots in Figure 8. It would better if the color could be consistent across these two figures. I found there are only 9 vegetation mixture categories in figure 9 while there are 13 categories in figure 8. Can you please explain this discrepancy? Also, I found some further inconsistency between these two figures. For example, the “Eucalyptus” in figure 9 corresponds to “Papyrus, Eucalyptus trees & Forest fragment” in figure 8 type 2 as they have the same spatial distribution. I am wondering why the categories are not consistent across these two figures.
4. Some sentences need to be revised to correct for potential grammatical errors and mistakes:

- “These three scales of wetland monitoring if consistently utilized, have the potent cost-effective effective and efficient”.
- b. “ In addition, this will create a record of long-term monitoring that will become an invaluable vital reference longer-term variations can be detected.”
- “Map developed our study clearly shows that a large demographic of urban dwellers are using wetlands for food security and poverty eradication.”
- “Labels of classes used in this study included broad categories of land use and land cover, agriculture, forest, wetlands, and agriculture in wetlands ( Figure 3), built up and bare ground”.

Is the work clearly and accurately presented and does it cite the current literature?

Yes

If applicable, is the statistical analysis and its interpretation appropriate?

Yes

Are all the source data underlying the results available to ensure full reproducibility?

No

Is the study design appropriate and is the work technically sound?

Yes

Are the conclusions drawn adequately supported by the results?

Yes

Are sufficient details of methods and analysis provided to allow replication by others?

Partly

Reviewer Expertise:

Remote sensing

I confirm that I have read this submission and believe that I have an appropriate level of expertise to confirm that it is of an acceptable scientific standard, however I have significant reservations, as outlined above.

# Reviewer report: Reviewer response for version 2

**DOI:** 10.21956/aasopenres.14490.r29130

**Published:** 2022-02-23

**Authors:** Patricia Dale

**Affiliations:** 1 Environmental Futures Research Institute (EFRI), School of Environment & Science, Griffith University, Nathan, Queensland, Australia

I have read the revised Kabiri *et al. *paper against my original comments and the summary of revisions.

 I am favourably impressed with the revision. It has addressed all the points satisfactorily and the paper is much improved and suitable for indexing. I have no further comments to make.

Is the work clearly and accurately presented and does it cite the current literature?

Partly

If applicable, is the statistical analysis and its interpretation appropriate?

Yes

Are all the source data underlying the results available to ensure full reproducibility?

Yes

Is the study design appropriate and is the work technically sound?

Yes

Are the conclusions drawn adequately supported by the results?

Yes

Are sufficient details of methods and analysis provided to allow replication by others?

Yes

Reviewer Expertise:

NA

I confirm that I have read this submission and believe that I have an appropriate level of expertise to confirm that it is of an acceptable scientific standard.

# Reviewer report: Reviewer response for version 1

**DOI:** 10.21956/aasopenres.14132.r28329

**Published:** 2021-02-08

**Authors:** Patricia Dale

**Affiliations:** 1 Environmental Futures Research Institute (EFRI), School of Environment & Science, Griffith University, Nathan, Queensland, Australia

The paper addresses an important issue: of wetlands and intensive use for urban agriculture. It shows wetland losses over a 3o year period using Landsat data and also classifies the recent characteristics using MODIS.  As it is an important piece of research, I recommend that the authors check my comments and edit the text to edit or clarify the points.

 There are some unclear parts (e.g why the 10000 NDVI values in Fig 7 and reference to land use and cover classes in Fig 3 which is simply 2 images with no indication of the classes).#

 It is difficult to clearly indicate issues without line numbers so I have marked comments in the pdf which I will attach here.

Is the work clearly and accurately presented and does it cite the current literature?

Partly

If applicable, is the statistical analysis and its interpretation appropriate?

Yes

Are all the source data underlying the results available to ensure full reproducibility?

Yes

Is the study design appropriate and is the work technically sound?

Yes

Are the conclusions drawn adequately supported by the results?

Yes

Are sufficient details of methods and analysis provided to allow replication by others?

Yes

Reviewer Expertise:

Wetland ecology, remote sensing

I confirm that I have read this submission and believe that I have an appropriate level of expertise to confirm that it is of an acceptable scientific standard, however I have significant reservations, as outlined above.

# Reviewer report: Reviewer response for version 1

**DOI:** 10.21956/aasopenres.14132.r27516

**Published:** 2020-07-30

**Authors:** Bjorn Brooks

**Affiliations:** 1 North Carolina Institute for Climate Studies, North Carolina State University, Asheville, NC, USA

The paper "Detecting level of wetland encroachment for urban agriculture in Uganda using hyper-temporal remote sensing" represents timely and important research in the area of environmental remote sensing, specifically being applied to land cover classification and land-use change.  The main premise employed is to extend and extrapolate field-based surveys on land cover types (crops) using remote sensing to canvas all land within the study domain. Specifically, two separate RS classification processes were completed: 1) to determine the amount of wetland loss between 1986 and 2016 using 2 different Landsat missions and 2) to determine the crop composition (map crop types) in 2016 using a 6-month time series of MODIS data.

 Overall I enjoyed the article but in its present form, I do not feel it can be indexed. I suggest provisionally accepting the article with the requirement of major revisions to the Results section (in particular "Characterization of crop agriculture in wetlands") such that the authors have specifically described how they developed the 50 phenology profile classes, as well as how exactly those 50 classes were clustered. Without those changes, the results presented in this paper are without concrete meaning.

 I have a number of additional specific changes to recommend. However, seeing that there are no line numbers to allow me to make specific references to issues, I have made specific comments and edits in a PDF file which can be found here .

 If the authors make these changes I strongly feel that all of my "partly" responses to the assessment questions above would become "yes". It is a nice paper if the authors wish to put in the additional work to bring it's printed presentation up to the same level as their original work effort.

Is the work clearly and accurately presented and does it cite the current literature?

Partly

If applicable, is the statistical analysis and its interpretation appropriate?

Partly

Are all the source data underlying the results available to ensure full reproducibility?

Partly

Is the study design appropriate and is the work technically sound?

Partly

Are the conclusions drawn adequately supported by the results?

Partly

Are sufficient details of methods and analysis provided to allow replication by others?

Partly

Reviewer Expertise:

Environmental remote sensing, geospatial statistics, landscape ecology, landscape dynamics, disturbance

I confirm that I have read this submission and believe that I have an appropriate level of expertise to confirm that it is of an acceptable scientific standard, however I have significant reservations, as outlined above.

# Response

**Published:** 2020-07-31

**Authors:** Stella Kabiri

**Affiliations:** Mukono Zonal Agricultural Research and Development Institute, Uganda

Dear Editor,

 On behalf of my co-authors and I, we are grateful for the time taken by the reviewer to review our manuscript and appreciate the suggestions made. We shall look at them carefully and update our manuscript based on the suggestions given to improve its scientific quality of the manuscript.

 Your sincerely,

 Dr. Stella Kabiri
